# Understanding and predicting the potency of ROS-based enzyme inhibitors, exemplified by naphthoquinones and ubiquitin specific protease-2†
†Electronic supplementary information (ESI) available: All experimental procedures, analytical data for small molecules. See DOI: 10.1039/c6sc02758j


**DOI:** 10.1039/c6sc02758j

**Published:** 2016-08-05

**Authors:** Pushparathinam Gopinath, Atif Mahammed, Shimrit Ohayon, Zeev Gross, Ashraf Brik

**Affiliations:** a Schulich Faculty of Chemistry , Technion-Israel Institute of Technology , Haifa , 3200008 , Israel . Email: chr10zg@tx.technion.ac.il ; Email: abrik@technion.ac.il

## Abstract

Recent studies have suggested that selective targeting of overexpressed enzymes in cancer cells by small molecules that induce the formation of reactive oxygen species (ROS) could be a viable approach in cancer therapy. One such example is the inactivation of ubiquitin specific protease-2 (USP2)—an emerging drug target to combat prostate cancer—by β-lapachone, which has been identified to involve oxidation of the catalytic cysteine's thiol residue to sulfinic acid. A rational design of β-lapachone analogs with improved activity requires a much better understanding of the variables that determine ROS production by this class of molecules. This crucial aspect was addressed *via* modulation of its 1,2-naphthoquinone scaffold and establishment of a structure/activity relationship, regarding its ability to reduce molecular oxygen to a ROS. The same series of compounds was also examined in terms of their inhibitory effect on the enzymatic activity of USP2. One deduction from these investigations was that the *ortho*-quinone motif in β-lapachone is much better suited for the catalytic reduction of oxygen than the *para*-quinone motif and some approved quinone based drugs. A broader conclusion, obtained from the series of compounds with *ortho*-quinone motifs, is that only the agents whose redox potential is in the narrow range of –0.3 ± 0.1 V (*vs.* Ag/AgCl in pH 7.5 aqueous buffer) induce the formation of ROS. The excellent correlation between the ROS production ability and the USP2 inhibition potency emphasizes that the relatively easy, fast, and reliable testing of electrocatalytic oxygen reduction by small molecules might be applied to screening and evaluating new drug candidates for similar targets.

## Abstract 2

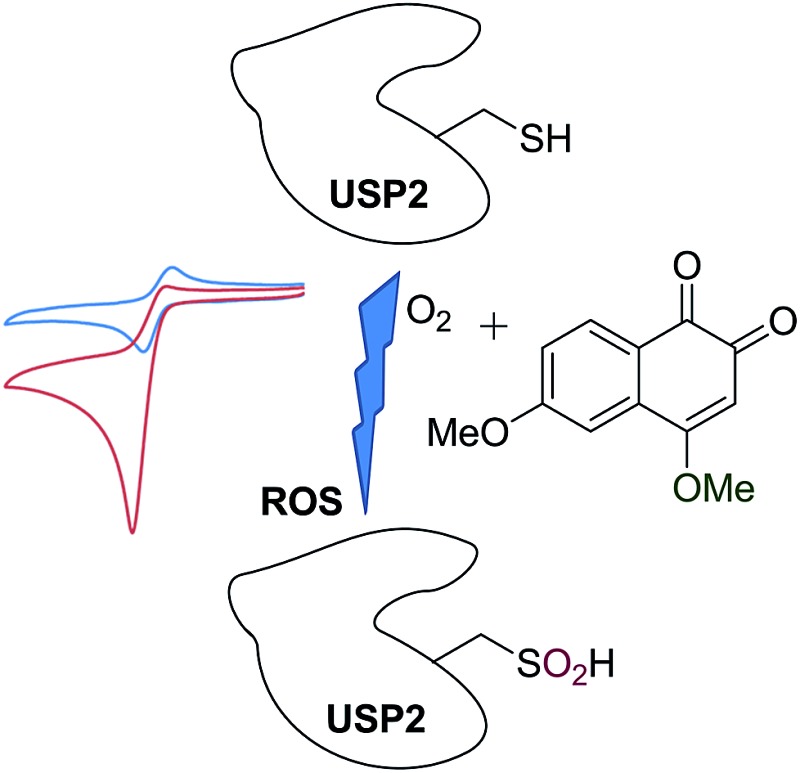
A multidisciplinary approach, composed of organic synthesis, electrochemistry, electrocatalysis and cellular studies, for correlating the molecular features of a 1,2-naphthoquinone scaffold with its ROS generating ability.

## Introduction

**Fig. 1:**
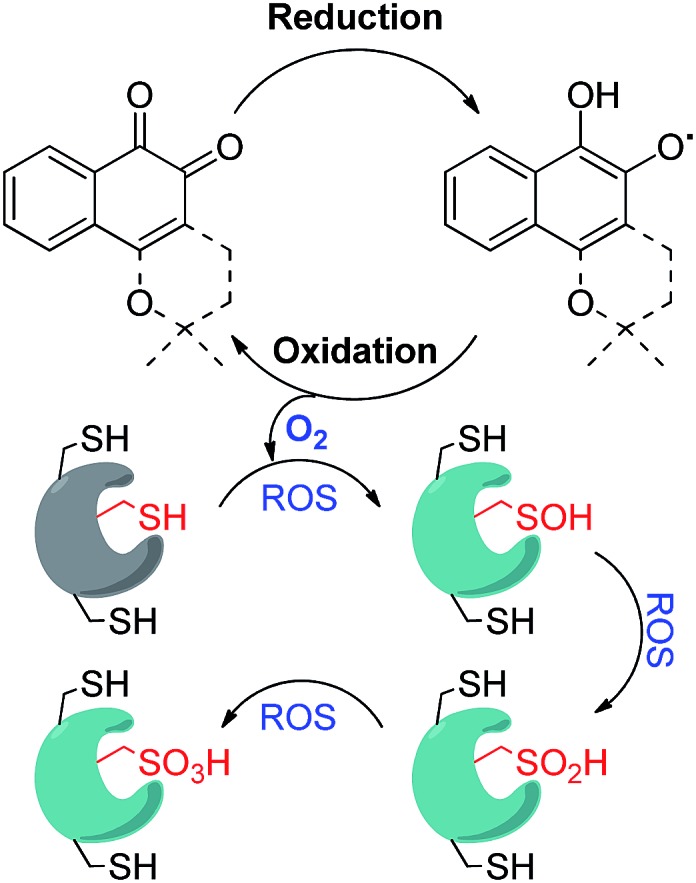
Schematic representation of redox cycling by *ortho*-quinones and their mode of inhibition of DUBs *via* oxidation of the catalytic Cys moiety mainly to sulfinic acid.

Reactive oxygen species (ROS) homeostasis is important for the survival and progression of both normal and cancerous cells.^1^ Certain amounts of ROS are required for proper cell function, including normal metabolism and signaling, but excessive amounts lead to oxidative stress—an imbalance between the production of ROS and their elimination by molecules or enzymes with antioxidant activity. Extreme oxidative stress will certainly lead to complete cell death, as in the case of treatment of tumors by photodynamic therapy (PDT),^2^ but the effect of mild conditions is much less predictable. The outcome depends very much on the primary target that will be modified by reacting with the ROS including lipids, DNA, proteins, particular enzymes, and more.^3^ While many cancer cells have developed mechanisms that assist in their survival under relatively high levels of ROS,^3^ they may still be vulnerable to exogenous small molecules that are known to generate ROS through redox cycling.^1^ This hypothesis has been supported by several recent studies, suggesting selective targeting of cancer cells with ROS-generating small molecules as a viable approach in cancer therapy.^4–6^ One class of cancer-relevant enzymes reported to be targeted by ROS are the cysteine proteases, whose catalytic Cys moiety has been found to undergo oxidation with consequential inhibition of their activities.^7^ The thiol of the catalytic Cys moiety may be oxidized to sulfenic acid (–SOH), sulfinic acid (–SO_2_H) or sulfonic acid (–SO_3_H), in a reversible manner in the first case and irreversible for the other two (Fig. 1).

Overexpression of the ubiquitination-counteracting deubiquitinases (DUBs), a subclass of cysteine proteases, is documented in several disease states like cancer, and neurodegenerative and viral diseases.^8,9^ Recent studies revealed that DUBs are susceptible to hydrogen peroxide, suggesting a potential way of regulating their cellular activity under oxidative stress (Fig. 1).^10–12^ For example, ubiquitin specific protease 1 (USP1) is connected with DNA damage repair, whereas the brain-abundant ubiquitin C-terminal hydrolase (UCHL-1) is linked to neurodegenerative diseases.^13^ DUBs are hence emerging as promising drug targets, and their targeting *via* a novel mechanism of inhibition has become a major goal in academia and in industry.^9^

We have recently reported the ROS-susceptibility of USP1 and ubiquitin specific protease 2 (USP2) by using the *ortho*-quinone natural product β-lapachone as a redox recycler.^14^ This molecule actually progressed up to phase II clinical trials for cancer treatment, and reported mechanisms of action included delay of the S-phase checkpoint in cancer cells^15^ and inhibition of NF-kB.^16^ We have contributed to this field by uncovering the effect of β-lapachone on DUBs, by demonstrating that the mechanism of inhibition by β-lapachone proceeds *via* ROS generation and irreversible oxidation of the catalytic Cys moiety to the sulfinic acid form (Fig. 1).^14^ Of particular interest is USP2, due to its association with aggressive prostate cancer and triple negative breast cancer.^17^ USP2 is associated with various known substrates in cells and affects the pathways that these substrates are involved in. The best-characterized substrate of USP2 is fatty acid synthase (FAS), responsible for protection of prostate cancer cells from apoptosis.^18^ The involvement of USP2 in various aspects of cancer survival leads to a great interest in the design and development of inhibitors against this DUB.

Realizing that β-lapachone is a ROS generating molecule for a defined target, *e.g.* USP2/1, prompted us to examine how changes in the *ortho*-quinone scaffold might modulate its redox potential and in turn affect its capacity to generate ROS, the consequences of DUBs inhibition and the cellular behavior of these inhibitors. Acquiring a structure/activity relationship profile and deducting the correlation with the redox properties might enable fine-tuning of potential inhibitors for therapeutic development. We now report a multidisciplinary approach, composed of organic synthesis, electrochemistry, electrocatalysis and cellular studies, for correlating the molecular features of the 1,2-naphthoquinone scaffold with its ROS generating ability. The results reveal large differences between the ROS-generating ability of *ortho*- *vs. para*-quinones, a very narrow window of redox potentials for ROS generation and an excellent relationship between ROS-generation and USP2 inhibition. Apoptosis induction by the lead compound (**12**) in DU145 cell lines is illustrated as well.

## Results

We initiated our study by preparing a focused set of 1,2-naphthoquinone derivatives based on the bicyclic core of β-lapachone, since it is the pharmacophore unit in this drug and such simplification enables us to rapidly access the desired compounds.

### (a) Synthesis

#### Synthesis of C4-substituted 1,2-naphthoquinones (**2–7**)

**Scheme 1:**
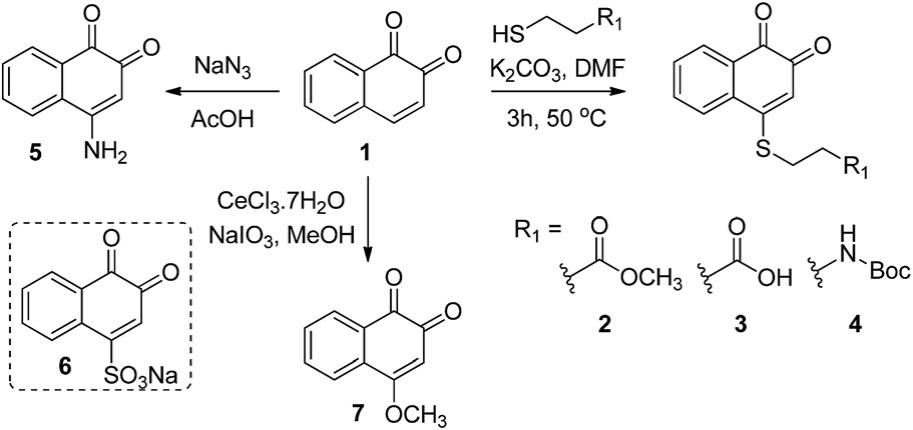
Synthesis of 1,2-naphthoquinones with different substituents on C4.

Upon facile Michael addition with methyl 3-mercaptopropionate (MMP), 3-mercaptopropionic acid (MPA), or 2-(Boc-amino)ethanethiol to the commercially available 1,2-naphthoquinone (**1**), compounds **2–4** were obtained, respectively, appended with the acid or the amine functionality suitable for further functionalization (Scheme 1). Direct incorporation of the amine onto the C4 position was achieved *via* reaction with sodium azide under acidic conditions, leading to compound **5**.^19^ 4-Methoxy-1,2-naphthoquinone (**7**) was obtained by the treatment of 1,2-naphthoquinone with methanol in the presence of equimolar CeCl_3_·7H_2_O and sodium iodate.^20^ Compound **6** is commercially available and was purchased from Acros Chemicals.

#### Synthesis of C5–C7-substituted 1,2-naphthoquinones (**8–14**)

**Scheme 2:**
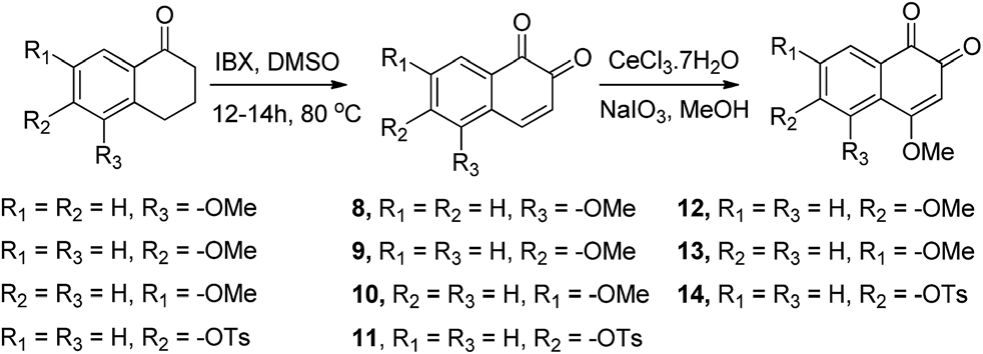
Synthesis of naphthoquinone derivatives (**8–14**) starting from tetralone derivatives.

Reaction of 5-, 6- and 7-methoxy and 6-OTs tetralones with 2-iodoxybenzoic acid (IBX) in DMSO at 80 °C afforded the corresponding 5-, 6- and 7-substituted 1,2 naphthoquinones **8–11**, respectively (Scheme 2).^21^ Reaction of compounds **9–11** with CeCl_3_·7H_2_O and sodium iodate in MeOH afforded the products **12–14** (Scheme 2, see the ESI† for experimental details).

### (b) USP2 inhibitions

**Fig. 2:**
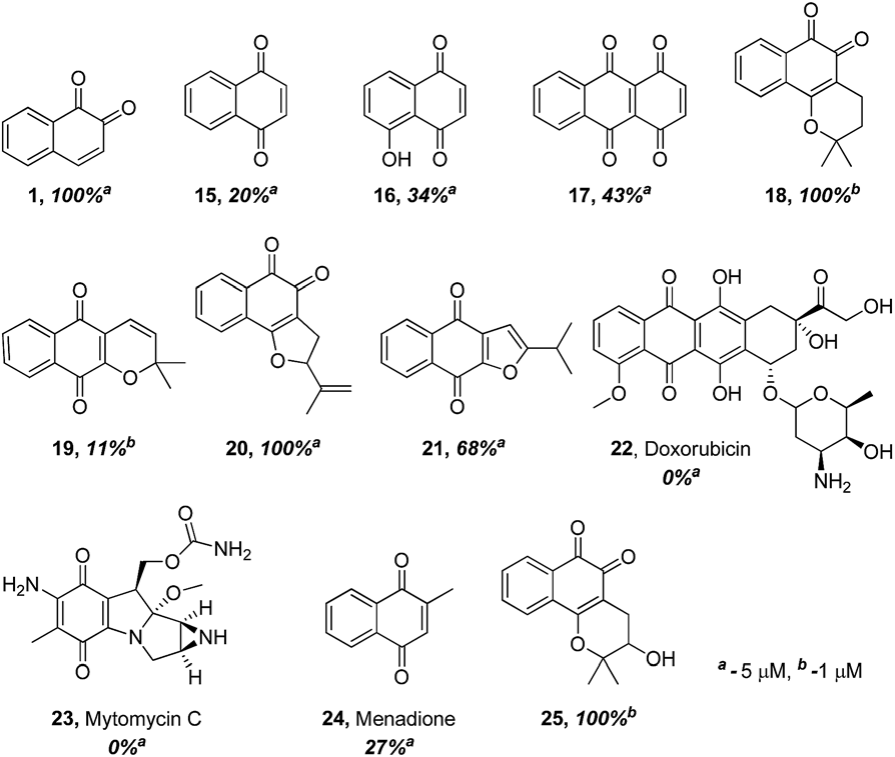
Comparison of USP2 inhibition capability of compounds with either *ortho*- or *para*-quinone moieties, at either 1 or 5 μM concentrations.

The finding that β-lapachone with its *ortho*-quinone moiety inhibits DUBs through ROS, prompted us to systematically investigate the effect of both *para*- and *ortho*-quinones against USP2 inhibition in addition to the synthesized *ortho*-quinone analogs.^22^ Towards this goal, a focused collection of quinone-containing molecules (**15–24**, Fig. 2) were obtained from commercial sources, which together with all the synthesized quinone derivatives described above, were tested for USP2 inhibition using our developed quenching pair assay.^23,24^ Among the non-substituted derivatives, *ortho*-naphthoquinone **1** exhibited full inhibition at 5 μM, while only 20% inhibition was obtained for the *para*-naphthoquinone counterpart **15** at the same concentration. Adding a hydroxyl substituent to give **16** or *para*-quinone to give **17** led to marginal improvements relative to the original compound **15**.^25^

The comparison between β-lapachone (**18**) and dehydro-α-lapachone (**19**) revealed complete inhibition against USP2 at 5 μM for both, however at 1 μM β-lapachone displayed 100% inhibition whereas the activity of dehydro-α-lapachone dropped to 11%. A similar comparison with the nor-β-lapachone (**20**) and nor-α-lapachone derivatives (**21**) disclosed 100% and 68% USP2 inhibition, respectively. Taken together, these results show that 1,2-quinones are consistently more potent USP2 inhibitors than 1,4-quinones.

Armed with these new findings, some selected anticancer drugs that are known to generate ROS [doxorubicin (**22**), mytomycin C (**23**) and menadione (**24**)] were screened to establish if DUBs are possible targets for them.^22^ These examinations revealed that compounds **22–24** did not show appreciable inhibition against USP2, even at 5 μM concentrations.

Compounds **2–7** (Scheme 1) have different substitutions on the C4 position of *ortho*-naphthoquinone **1**: *S*-alkyl groups in **2–4**, amine in **5**, SO_3_^–^ in **6**, and methoxy in **7**. Compounds **2–4** did not exhibit measurable activity against USP2 at 1 μM, which might be attributed to oxidation of the sulfide-moiety therein by the ROS. Compound **6** with its electron-withdrawing sulfonyl group did not show any inhibition at 1 μM, while compounds **5** and **7** with their electron-donating groups (–NH_2_ and –OCH_3_, respectively) exhibited substantially increased activity relative to the parent compound **1**. Here we observed 33% inhibition at 500 nM for **5** and nearly complete inhibitory activity at 400 nM for **7**. Taken together, the methoxy substituent in **7** led to an about 12-fold increase in the activity compared to the unsubstituted naphthoquinone **1**, which indicates that electron-donating groups provide a beneficial effect when presented on C4.

Compounds in which a methoxy group is present on the non-quinonic ring of 1,2-naphthoquinones, at positions 5, 6 and 7 (compounds **8–10**) were also prepared, however none of them displayed improved inhibitory activity at 1 μM. In contrast, compounds **12–14** which have C5- or C6-substituents in addition to the C4–OCH_3_, were potent inhibitors. In these cases, we observed 47% inhibition at 300 nM for **12**, 28% at 300 nM for **13**, and 32% inhibition at 500 nM for **14**. 3-Hydroxy β-lapachone (**25**, Fig. 2)^26^ exhibited 78% inhibition at 300 nM, and was the best candidate in the tricyclic class of compounds.

**Fig. 3:**
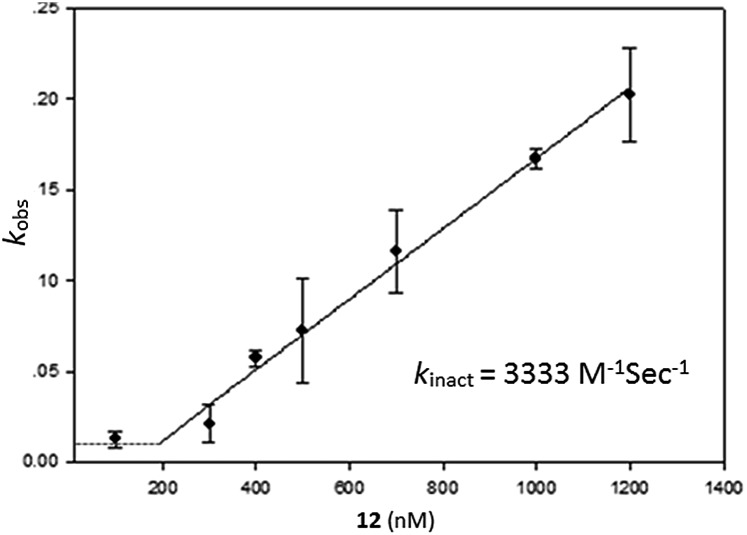
Plot of the inactivation rate constants (*k*_obs_) *vs.* the concentration of **12** (nM), for obtaining the maximal rate of enzyme inactivation (*k*_inact_). Each value represents the mean ± SE of two independent experiments.

Having identified compound **12** as the most potent bicyclic inhibitor, its *k*_inact_ was determined and found to be 3333 M^–1^ s^–1^ (Fig. 3). To verify that **12** also inhibited USP2 *via* the oxidation mechanism proposed for β-lapachone, the mass of the enzyme was measured before and after treatment with compound **12**. The 32 Da increase measured is in perfect agreement with the conversion of the catalytic Cys to sulfinic acid (Fig. 1, ESI†).

### (c) Electrochemistry of naphthoquinone derivatives

Having measured the inhibition of USP2 with our focused library of quinone derivatives, we then focused our attention on their electrochemical behavior in an attempt to correlate the enzyme inhibition activity with their ROS-generating capabilities.

#### Electrochemistry under an inert atmosphere

The cyclic voltammograms (CV) of the naphthoquinone compounds were recorded under a nitrogen environment in both organic and aqueous solutions. The reduction potentials were deduced to be –0.67, –0.71, and –0.68 V in acetonitrile and –0.23, –0.24, and –0.24 V in Tris buffer of pH 7.5, for menadione (**24**), β-lapachone (**18**) and dehydro-α-lapachone (**19**), respectively.

**Fig. 4:**
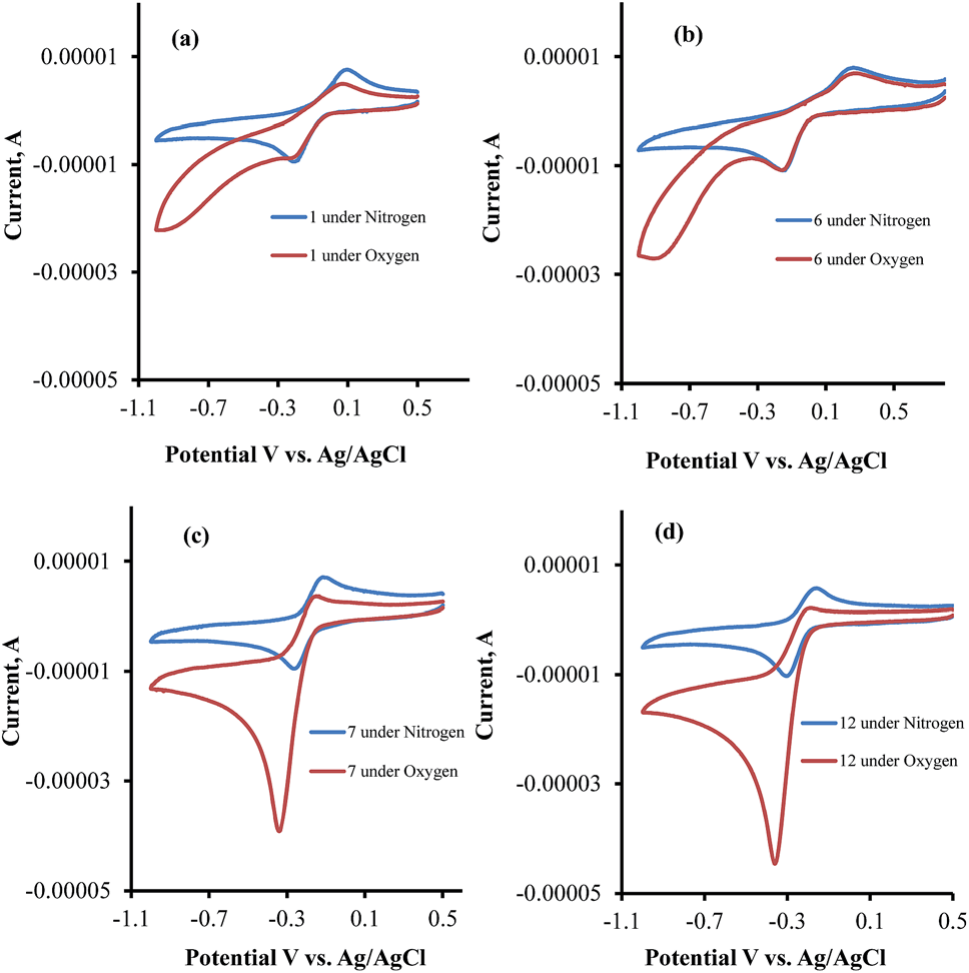
Cyclic voltammograms of (a) **1**, (b) **6**, (c) **7** and (d) **12** in Tris buffer under nitrogen and oxygen atmospheres.

To understand the influence of electron-donating and -withdrawing substituents on C4 of 1,2-naphthaquinone on the reduction potential, the CV of compounds **1** and **5–7** were examined in acetonitrile solution (Fig. 4). This study revealed that the substitution of the naphthoquinone with the electron-withdrawing SO_3_Na group (**6**) induced a positive shift of the reduction potential (easier to be reduced by 180 mV, Fig. 4b) while substitution with the electron-donating OCH_3_ group (**7**) or NH_2_ group (**5**) shifted the reduction potential in the negative direction (harder to be reduced by 160 mV for **7**, Fig. 4c, and by 160 mV for **5**). Similar results were obtained in Tris buffer, pH 7.5.^27^

CV examinations of compounds **9**, **12**, and **14** were also performed in acetonitrile solution and compared to those of **1** and **7**. The influence of electron-donating and -withdrawing substituents on the aromatic ring of the 1,2-naphthoquinone on the reduction potential was deduced to be considerably less than that when present on the quinone moiety.^27^ Relative to **1**, the reduction potential of the C4–OCH_3_ compound (**7**) is shifted by –160 mV and that of the C6–OCH_3_ isomer (**9**) by only –50 mV. An additive effect of the substituents is obtained for the compound that contains two methoxy groups (**12**) whose reduction potential is shifted by –220 mV. On the other hand, the shift for the C4-methoxy-C6-tosylate-1,2-naphthoquinone (**14**) is only –60 mV, reflecting the simultaneous substitution of the 1,2-naphthoquinone building block by electron-donating and -withdrawing groups. Very similar trends were obtained for the same series of compounds, when their CV analyses were recorded in Tris buffer, pH 7.5.

#### Electrochemistry under an O_2_ atmosphere

**Fig. 5:**
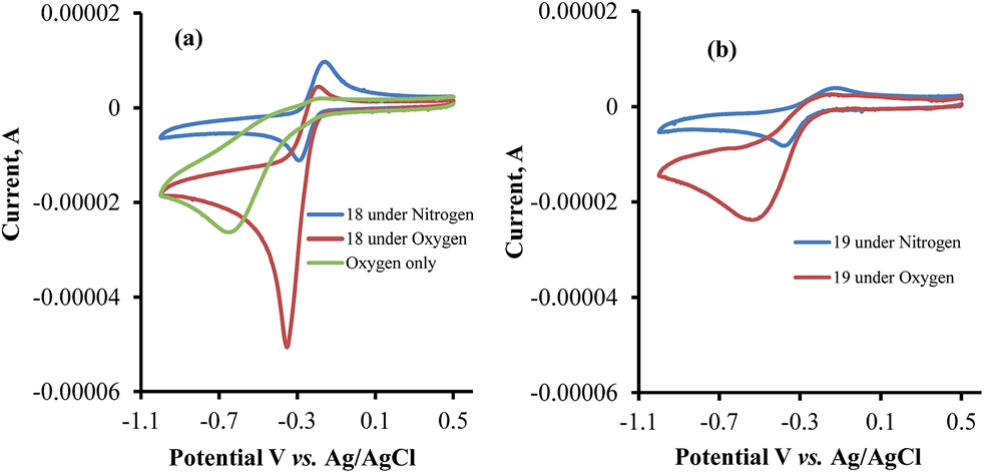
(a) Cyclic voltammograms of β-lapachone **18** in Tris buffer under nitrogen and oxygen atmospheres and that of the oxygen saturated solution without **18**. (b) Cyclic voltammograms of dehydro-α-lapachone **19** in Tris buffer under nitrogen and oxygen atmospheres.

The abovementioned CV's were also recorded in aqueous Tris buffer saturated with oxygen to examine any electrocatalytic reduction of oxygen by menadione, β-lapachone, or dehydro-α-lapachone. A catalytic cathodic current in the presence of oxygen was obtained for all compounds, testifying that the reduced naphthoquinones catalyze the reduction of oxygen to O_2_^–^˙, the precursor of all biologically relevant ROS. Since the chromatograms are reversible under nitrogen, the ratio between the cathodic and anodic currents (*i*_cat_/*i*_p_) obtained under oxygen becomes a criterion for the catalytic activity.^28^ This ratio was determined to be 4.7, 3.2, and 2.2 for β-lapachone, dehydro-α-lapachone, and menadione, respectively (Fig. 5). This difference clearly shows that β-lapachone catalyzes the reduction reaction of oxygen much more efficiently than its *para*-analogs. Since their redox potentials are practically identical, any biologically relevant reducing agent capable of reducing them will lead to a larger amount of ROS, in the case of β-lapachone, because of the higher *i*_cat_/*i*_p_ value. The stronger inhibitory activity of β-lapachone relative to dehydro-α-lapachone and menadione hence suggests that the larger potency of the former is due to more efficient production of the enzyme-damaging ROS.

The CV's of **1**, **6**, **7**, and **12** were also recorded under both N_2_ and O_2_ atmospheres (Fig. 4a–d). Compounds **7** and **12** show catalytic activities for oxygen reduction, while **1** and **6** do not. The (*i*_cat_/*i*_p_) for all the naphthoquinones that were studied in this work are summarized in Table 1.

**Table 1:** USP2 inhibition, redox potentials in volts, and catalytic oxygen reduction ability of the naphthoquinone derivatives

Compound	% USP2 inhibition, concentration				*E*_1/2_ ^*a*^	*E*_1/2_ ^*b*^	*i*_cat_/*i*_p_ ^*c*^	*E* at *i*_cat_	Δ*E* ^*d*^
	1000 nM	500 nM	400 nM	300 nM	CH_3_CN	H_2_O	H_2_O	H_2_O	
**25**	100	96	96	78	–0.72	–0.24	4.9	–0.39	0.15
**18**	100	100	85	19	–0.71	–0.24	4.7	–0.36	0.12
**12**	100	95	94	47	–0.72	–0.23	4.6	–0.35	0.12
**7**	100	100	93	33	–0.66	–0.20	4.6	–0.32	0.12
**5**	100	33	—	—	–0.66	–0.30	3.7	–0.48	0.18
**9**	0	0	—	—	–0.55	–0.11	2.0	–0.40	0.29
**14**	100	32	0	0	–0.56	–0.15	1.8	–0.40	0.25
**1**	0	—	—	—	–0.50	–0.06	1.0	—	—
**6**	0	0	—	—	–0.32	+0.06	1.0	—	—

***para*-Naphthoquinones**									
**19**	11	—	—	—	–0.68	–0.24	3.2	–0.53	0.29
**24**	0 at 0.5 μM	—	—	—	–0.67	–0.23	2.2	–0.42	0.19

^*a*^ *V vs.* SCE, ∼0.4 mM compound, 0.1 M TBAP, in CH_3_CN under N_2_.

^*b*^ *V vs.* SCE, ∼0.4 mM compound, aq. Tris buffer, pH 7.5, under N_2_.

^*c*^∼0.4 mM compound, aq. Tris buffer, pH 7.5, under O_2_.

^*d*^ *E*_1/2_ – *E* at *i*_cat_, both in Tris buffer, pH 7.5.

### (d) Cell study

**Fig. 6:**
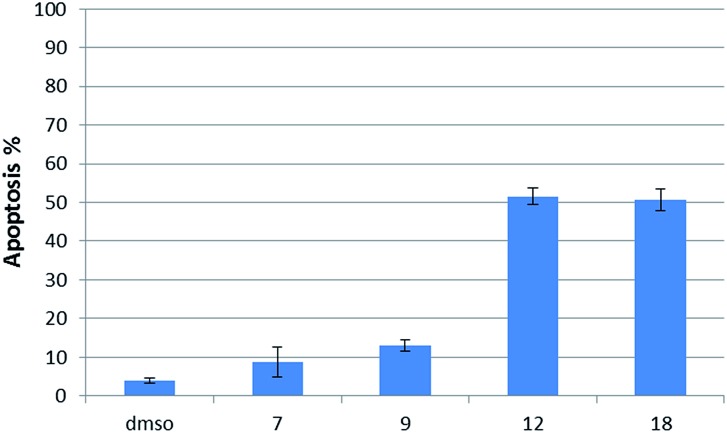
Apoptosis level in DU145 cells treated with **7**, **9**, **12** and **18** for 2 h using an annexin V-FITC apoptosis detection kit (BD Biosciences) according to the manufacturer's protocol and monitored *via* flow-cytometry.

Having several potent bicyclic quinones in hand, we checked the ability of compounds **7**, **9**, **12** and **18** to induce apoptosis in DU145 prostate cancer cells, in which USP2 is overexpressed.^14^ Incubation at 6 μM concentration for two hours resulted in 3.92% apoptosis for the DMSO control, 50% for β-lapachone, ∼51% for **12**, 13% for **9** and 9% for **7** (Fig. 6).

## Discussion

The ability of the *para*-quinone based anticancer drugs (*e.g.* menadione) to generate ROS and our earlier finding that β-lapachone with its *ortho*-quinone moiety inhibits DUBs through ROS induced damage to the enzyme, prompted us to systematically investigate the effect of both *para*- and *ortho*-naphthoquinones against USP2 inhibition.^22,25^ Towards the above goal, a focused collection of quinone-containing molecules (Fig. 2 and Schemes 1 and 2) were tested for USP2 inhibition. The investigations started with a comparison between the non-substituted *ortho*-naphthoquinone **1** and various *para*-naphthoquinones: **15–17** and the anticancer drugs **22–24**.^22^ The apparent superior inhibitory effect of **1** relative to these six compounds triggered efforts towards the synthesis of substituted 1,2-naphthoquinones, of which three (**25**, **12**, and **7**) were identified to be more potent USP2 inhibitors than β-lapachone.

In the search for the origin of the superiority of *ortho*- *vs. para*-naphthoquinones, both the reduction potentials (quinone/semiquinone radical, determined under anaerobic conditions) and the electrocatalytic activity for reduction of oxygen (to O_2_^–^˙, which undergoes spontaneous disproportionation to H_2_O_2_ and O_2_) were determined for 11 derivatives. This disclosed that in all cases of identical reduction potentials, the catalytic activity (displayed in terms of *i*_cat_/*i*_p_) of *ortho*-quinones very much exceeds that of analogous *para*-quinones. This is apparent from the results summarized in Table 1, wherein the reduction potentials of compounds **19** and **24** are practically identical (between –0.20 and –0.24 V in aqueous pH 7.5 buffer) to those of **7**, **12**, **18**, and **25**. However the two *para*-quinone derivatives (**19** and **24**) are much less efficient O_2_ reduction catalysts. The latter phenomenon is not only apparent from the lower *i*_cat_/*i*_p_ ratios, but also from the difference between the voltage of maximum catalytic current and the *E*_1/2_ values (Δ*E* in Table 1).

The data obtained regarding electrocatalytic activity serves well for addressing a reoccurring puzzle presented in many literature reports: how organic molecules that are reduced more easily^29,30^ (*i.e.* at less negative redox potentials) than molecular oxygen can still catalyze the reduction of the latter? Under the present conditions (aqueous buffer solution of pH = 7.5), the reduction potential *vs.* Ag/AgCl of β-lapachone under N_2_ atmosphere is –0.24 V, while that of dissolved oxygen in the absence of β-lapachone is –0.53 V (–0.33 *vs.* NHE).^31^ Still, examination of the chromatogram of β-lapachone under an oxygen atmosphere (compound **18**, Fig. 5a) clearly reveals that the reduction of oxygen becomes more efficient (indicated by the larger current) and appears at a much less negative potential (maximal at –0.36 V) under these conditions. In fact, the coinciding of the voltage for maximum current in the absence and presence of oxygen clearly testifies that β-lapachone acts as a true electrocatalyst. An identical type of examination for dehydro-α-lapachone (compound **19**, Fig. 5b) shows that this isomer is much less potent regarding both terms: the catalytic current (relatively low *i*_cat_/*i*_p_) and almost no shift to lower overpotential (maximal at –0.53 V as without the catalyst). The catalytic activity of menadione (**24**) is even smaller.

**Scheme 3:**
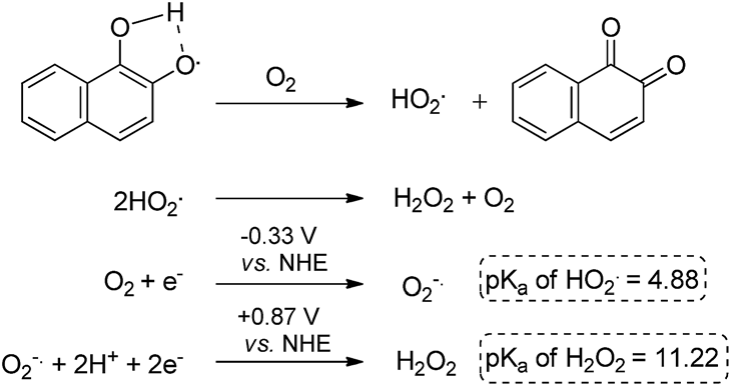
Possible mechanism of hydrogen peroxide generation.

A reasonable explanation for the larger catalytic activity of *ortho*-quinone relative to *para*-quinone for reducing oxygen might be attributed to the stability of the one-electron reduction product obtained in neutral solution, a semiquinone radical.^32^ The *ortho*- but not *para*-semiquinone radical intermediate may be stabilized by hydrogen bonding of the vicinal oxygen atoms and a proton, *via* a five-membered ring (Scheme 3).^33–37^ The acidity of this trapped proton should be taken into account when analyzing the reaction with oxygen, by two means: (a) it may induce an electron-coupled proton transfer to produce HO_2_˙ rather than ionized O_2_^–^˙; and (b) it may facilitate the subsequent reduction to hydrogen peroxide (Scheme 3).^31,38^ On the other hand, reduction of the *para*-quinone in neutral water solution will produce the non-stabilized semiquinone radical intermediate, which can only reduce oxygen *via* an electron transfer. The produced superoxide anion radical will be relatively stable regarding the second reduction to H_2_O_2_, until it reacts with a proton from the solution to produce a protonated superoxide radical. In simple words, the *ortho*-semiquinone radical intermediate may induce a general acid catalytic effect for the reduction of O_2_, while catalysis by the *para*-semiquinone radical intermediate proceeds only *via* specific acid catalysis.

**Fig. 7:**
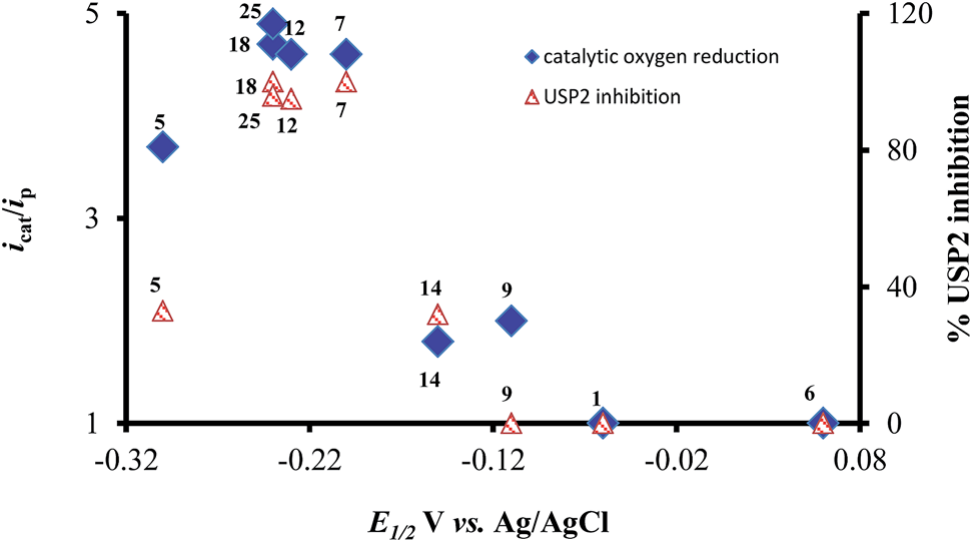
Correlation between reduction potentials of *ortho*-naphthoquinones, catalytic reduction currents of oxygen, and the % USP2 inhibition at 500 nM of the *ortho*-naphthoquinones.

The most interesting result of the investigations is the correlation between the redox potentials of the *ortho*-naphthoquinones, their electrocatalytic activity, and their ability to serve as inhibitors of USP2. The results of Table 1 clearly show that the potent inhibitors are very active catalysts for oxygen reduction and that the window of opportunity in terms of the quinone/semiquinone redox potentials is very narrow. The interpretation is that compounds that undergo reduction at potentials lower (more negative) than –0.3 V (*vs.* SCE, at pH 7.5) might be too short-lived to induce the bimolecular reaction with oxygen (kinetic considerations), while the reducing power of those that are reduced at potentials higher than –0.1 V is too low regarding electron transfer to oxygen (thermodynamic considerations). Even more appealing is the almost perfect correlation between the electrocatalytic activity of the *ortho*-naphthoquinones and USP2 inhibition, which is further demonstrated in Fig. 7. The only exception is compound **5**, which according to Fig. 7 and the data in Table 1 should be quite a poor inhibitor. This particular compound however contains a C4–NH_2_ group which may undergo oxidation or protonation, or participate in H-bonding as both a H-donor and a H-acceptor, and these features may significantly differ in pure aqueous and protein-containing media. These variables may affect both the inhibitory effects and electrocatalysis, which is apparently the reason for its exceptional behavior.

The examination of DU145 prostate cancer cells, in which USP2 is overexpressed, regarding induced cytotoxicity *via* treatment with five selected quinones (Fig. 6) disclosed that only compound **12** was (marginally) more potent than β-lapachone (**18**). This result and the low potency of compound **9** are consistent with their independently acquired information regarding USP2 inhibition, redox potentials, and ROS generation. On the other hand, the same kind of rather naïve analysis would lead to the expectation that compounds **25** and **7** should also be very cytotoxic, which is clearly not the case. There are many possible reasons for that shortcoming, however these are out of the scope of the present investigations.^39^ There is still no doubt that ROS generation affects the enzymatic activity of USP2, but in more realistic systems there are many more targets for those ROS and their identities might change as a function of the closeness of the particular ROS-generating molecule (naphthoquinones in the present case) to them.

## Conclusions

Understanding the parameters that govern ROS generation by small molecules is crucial for the design of efficient inhibitors for biological targets. In this work, we systematically investigated the effect of substituents on the 1,2-naphthoquinone scaffold for beneficial USP2 inhibition. Specifically, our studies on the quinone/semiquinone redox potentials, and the electrocatalytic reduction of molecular oxygen uncovered very meaningful structure/activity relationships. The comparison of 1,2- and 1,4-naphthoquinone derivatives with identical quinone/semiquinone redox potentials revealed that the former compounds were invariably more potent enzyme inhibitors as well as better electrocatalysts. The latter feature was attributed to a hydrogen-bonding network present in the *ortho*-semiquinone radicals, which provides the opportunity of general acid catalysis for the reduction of oxygen. Formation of reduced oxygen, the precursor of all ROS, was most significant for compounds within a very narrow range of redox potentials. Optimization of all deduced variables led to the identification of a new lead compound with beneficial USP2 inhibition and redox properties: the 4-methoxy-substituted 1,2-naphthoquinone (**12**). The obtained lead compound **12** possesses a simplified structure compared to β-lapachone, and yet exhibited potent inhibition of USP2 activity. Notably, the effect of substituents on the quinone ring is more influential on the inhibition of USP2 compared to substitutions on the aromatic ring. In addition, we also demonstrated that the mode of inhibition of **12** is through the oxidation of a catalytic Cys moiety to its sulfinic acid state and further showed that it induces apoptosis in DU145 cells. Altogether, this study uncovers an efficient strategy that may be applied in other systems that are affected by the generation of ROS.

## References

[cit1] Trachootham D., Alexandre J., Huang P. (2009). Nat. Rev. Drug Discovery.

[cit2] Hopper C. (2000). Lancet Oncol..

[cit3] Liou G.-Y., Storz P. (2010). Free Radical Res..

[cit4] Raj L., Ide T., Gurkar A. U., Foley M., Schenone M., Li X., Tolliday N. J., Golub T. R., Carr S. A., Shamji A. F., Stern A. M., Mandinova A., Schreiber S. L., Lee S. W. (2011). Nature.

[cit5] Trachootham D., Zhou Y., Zhang H., Demizu Y., Chen Z., Pelicano H., Chiao P. J., Achanta G., Arlinghaus R. B., Liu J., Huang P. (2006). Cancer Cell.

[cit6] Shaw A. T., Winslow M. M., Magendantz M., Ouyang C., Dowdle J., Subramanian A., Lewis T. A., Maglathin R. L., Tolliday N., Jacks T. (2011). Proc. Natl. Acad. Sci. U. S. A..

[cit7] Paulsen C. E., Carroll K. S. (2013). Chem. Rev..

[cit8] Komander D., Clague M. J., Urbe S. (2009). Nat. Rev. Mol. Cell Biol..

[cit9] Gopinath P., Ohayon S., Nawatha M., Brik A. (2016). Chem. Soc. Rev..

[cit10] Cotto-Rios X. M., Bekes M., Chapman J., Ueberheide B., Huang T. T. (2012). Cell Rep..

[cit11] Kulathu Y., Garcia Francisco J., Mevissen Tycho E. T., Busch M., Arnaudo N., Carroll Kate S., Barford D., Komander D. (2013). Nat. Commun..

[cit12] Lee J.-G., Baek K., Soetandyo N., Ye Y. (2013). Nat. Commun..

[cit13] Maraganote D. M., Lesnick T. G., Elbaz A., Charrier-Harlin M.-C., Gasser T., Krueger R., Hattori N., Mellick G. D., Quattrone A., Satoh J.-i., Toda T., Wang J., Ioannidis J. P. A., de Andrade M., Rocca W. A. (2004). Ann. Neurol..

[cit14] Ohayon S., Refua M., Hendler A., Aharoni A., Brik A. (2015). Angew. Chem., Int. Ed..

[cit15] Li Y., Sun X., LaMont J. T., Pardee A. B., Li C. J. (2003). Proc. Natl. Acad. Sci. U. S. A..

[cit16] Rodrigues de Almeida E. (2009). Open Nat. Prod. J..

[cit17] Qu Q., Mao Y., Xiao G., Fei X., Wang J., Zhang Y., Liu J., Cheng G., Chen X., Wang J., Shen K. (2015). Tumor Biol..

[cit18] Priolo C., Tang D., Brahamandan M., Benassi B., Sicinska E., Ogino S., Farsetti A., Porrello A., Finn S., Zimmermann J., Febbo P., Loda M. (2006). Cancer Res..

[cit19] Fieser L. F., Hartwell J. L. (1935). J. Am. Chem. Soc..

[cit20] Takuwa A., Soga O., Iwamoto H., Maruyama K. (1986). Bull. Chem. Soc. Jpn..

[cit21] Ren J., Lu L., Xu J., Yu T., Zeng B.-B. (2015). Synthesis.

[cit22] Soares K. M., Blackmon N., Shun T. Y., Shinde S. N., Takyi H. K., Wipf P., Lazo J. S., Johnston P. A. (2010). Assay Drug Dev. Technol..

[cit23] Ohayon S., Spasser L., Aharoni A., Brik A. (2012). J. Am. Chem. Soc..

[cit24] Ohayon S., Refua M., Brik A. (2015). Org. Biomol. Chem..

[cit25] Dharmaraja A. T., Chakrapani H. (2014). Org. Lett..

[cit26] 3-Hydroxy β-lapachone was synthesized starting from lapachol and the experimental detail is presented in the ESI.

[cit27] Fieser L. F., Fieser M. (1935). J. Am. Chem. Soc..

[cit28] *i* _cat_ is the catalytic current measured in the presence of O_2_ and *i* _p_ is the peak current measured in the absence of O_2_. The *i* _cat_/*i* _p_ ratio reflects the kinetics for catalyzing the reduction of oxygen and hence the kinetics for producing ROS. The higher the *i* _cat_/*i* _p_ ratio obtained for a naphthoquinone derivative the faster it will catalyze the reduction of oxygen for producing ROS

[cit29] Nasiri H. R., Bolte M., Schwalbe H. (2008). Nat. Prod. Res..

[cit30] Brisach-Wittmeyer A., Souna Sido A. S., Guilini P., Desaubry L. (2005). Bioorg. Med. Chem. Lett..

[cit31] Bielski B. H. J., Cabelli D. E., Arudi R. L., Ross A. B. (1985). J. Phys. Chem. Ref. Data.

[cit32] Klod S., Dunsch L. (2011). Magn. Reson. Chem..

[cit33] Abreu F. C., Goulart M. O. F., Brett A. M. O. (2002). Electroanalysis.

[cit34] Frontana C., Gonzalez I. (2005). J. Braz. Chem. Soc..

[cit35] Ci X., Silveira da Silva R., Nicodem D., Whitten D. G. (1989). J. Am. Chem. Soc..

[cit36] Alegria A. E., Sanchez-Cruz P., Rivas L. (2004). Free Radical Biol. Med..

[cit37] Villamil S. F., Stoppani A. O. M., Dubin M. (2004). Methods Enzymol..

[cit38] Wood P. M. (1988). Biochem. J..

[cit39] Adams D. J., Boskovic Z. V., Theriault J. R., Wang A. J., Stern A. M., Wagner B. K., Shamji A. F., Schreiber S. L. (2013). ACS Chem. Biol..

