# Supplementary material for: Understanding and predicting the potency of ROS-based enzyme inhibitors, exemplified by naphthoquinones and ubiquitin specific protease-2
Source: Chem Sci. 2016 Aug 5;7(12):7079–86. doi: 10.1039/c6sc02758j (PMC5355956; doi:10.1039/c6sc02758j)
Supplement: Supplementary file 1 [file SC-007-C6SC02758J-s001.pdf]

Electronic Supplementary Material (ESI) for Chemical Science.
This journal is © The Royal Society of Chemistry 2016

Understanding and predicting the potency of ROS-based enzyme inhibitors, exemplified by naphthoquinones and ubiquitin specific protease-2

Pushparathinam Gopinath,# Atif Mahammed,# Shimrit Ohayon, Zeev Gross* and Ashraf Brik*

Schulich Faculty of Chemistry, Technion-Israel Institute of Technology Haifa, 3200008 (Israel)

chr10zg@tx.technion.ac.il

abrik@technion.ac.il

# these authors contributed equally

Materials and methods

General methods ^1H and ^{13}C NMR spectra were recorded using $CDCl_3$ and DMSO-d_6 as a solvents. Chemical shifts were reported in δ units (ppm) with reference to TMS as an internal standard, and J values are given in Hz. ^1H and ^{13}C-NMR spectra were recorded on a Bruker AMX-400 MHz spectrometer. Mass determination of the materials was carried out using an LCQ Fleet Ion Trap (Thermo Scientific). Flash column chromatography was carried out with silica gel (220–440 mesh). The reactions were carried out in oven-dried glassware under nitrogen. Chemicals and compounds **1**, **6**, **15**, **16**, **17**, **18**, **19**, **20**, **21**, **22**, **23**, **24** were purchased from Aldrich, Fluka and Alfa Aesar. Commercial reagents were used without further purification. Analytical thin-layer chromatography (TLC) was performed on pre-coated plates (0.25 mm, silica gel 60 F254). Compound spots were visualized by UV light (254 nm).

Cyclic voltammetry (CV)

A WaveNow USB potentiostat Galvanostat (Pine Research Instrumentation) was utilized, using Pine After-Math Data Organizer software. A three electrode system was used, consisting of a mini glassy carbon electrode (diameter of the active zone: 2.8 mm; Metrohm) working electrode, a platinum wire counter electrode, and an Ag/AgCl reference electrode. The CV measurements in organic solvent were performed in acetonitrile solutions which contained 0.1 M in tetrabutylammonium perchlorate (TBAP, Fluka, recrystallized twice from methanol) and 0.4 mM substrate under N_2 atmosphere at ambient temperature. The $E_{1/2}$ value for the Ferrocene/Ferrocenium couple under these conditions was 0.47 V. The CV measurements in aqua solutions were performed in Tris buffer, pH 7.5 which contained 10% DMSO (for solubilizing the substrates) and 0.4 mM substrate under N_2 and O_2 atmosphere at ambient temperature. Scan rates of 10–1000 mV/s were applied.

Procedure for the preparation of compound 2:

To a stirred suspension of **1** (250mg, 1.58 mmol) and K_2CO_3 (1.30g, 9.49 mmol) in DMF (10 mL), methyl 3-mercaptopropionate (175 μl, 1.58 mmol) was added at room temperature and allowed to stir for 3h at 50 °C. TLC showed the complete disappearance of starting material. The reaction was quenched with water, extracted with EtOAc and purified by column using $CHCl_3$-EtOAc as eluents to give product **2**, as a dark red solid (312mg, 52% yield). ^1H NMR ($CDCl_3$) δ 8.09 (dd, *J* = 7.6, 1.2 Hz, 1H), 7.74 (d, *J* = 7.8 Hz, 1H), 7.61 (td, *J* = 7.7, 1.3 Hz, 1H), 7.51 (t, *J* = 7.5 Hz, 1H), 6.35 (s, 1H), 3.69 (s, 3H), 3.26 (t, *J* = 7.1 Hz, 2H), 2.77 (t, *J* = 7.1 Hz, 2H); ^{13}C

NMR ($CDCl_3$) δ 179.45, 176.37, 171.15, 158.89, 135.19, 133.55, 131.42, 130.58, 129.50, 125.31, 119.76, 52.40, 32.28, 26.45. HRMS (ESI) exact mass calcd. for $C_{17}H_9O_4$ $[M+H]^+$ 277.0501, found $[M+H]^+$ 277.0501.

Compound **3** (152mg, 37% yield) was prepared starting from **1** (250mg, 1.58 mmol) according to the procedure described above, which was isolated as a red solid. ^1H NMR (DMSO) δ 12.85–12.35 (bs, 1H), 8.05 – 7.97 (m, 1H), 7.85 – 7.74 (m, 2H), 7.66 (td, *J* = 7.4, 1.4 Hz, 1H), 6.45 (s, 1H), 3.34 (t, *J* = 6.8 Hz, 2H), 2.75 (t, *J* = 6.8 Hz, 2H); ^{13}C NMR (DMSO) δ 178.71, 175.74, 172.30, 156.73, 135.07, 133.09, 131.15, 130.57, 128.31, 124.92, 120.16, 32.10, 25.99. HRMS (ESI) exact mass calcd. for $C_{13}H_{10}O_4SNa$ $[M+Na]^+$ 285.0198, found $[M+H]^+$ 285.0138.

Compound **4** (280 mg, 53% yield) was prepared starting from **1** (250mg, 1.58 mmol) according to the procedure described above, which was isolated as a pale yellow solid. ^1H NMR ($CDCl_3$) δ 8.16 (dd, *J* = 7.6, 1.2 Hz, 1H), 7.84 (d, *J* = 7.8 Hz, 1H), 7.68 (td, *J* = 7.7, 1.3 Hz, 1H), 7.57 (t, *J* = 7.6 Hz, 1H), 6.46 (s, 1H), 4.96 (s, 1H), 3.53 (dd, *J* = 12.5, 6.2 Hz, 2H), 3.23 (t, *J* = 6.5 Hz, 2H), 1.46 (s, 9H).^{13}C NMR ($CDCl_3$) δ 179.39, 176.27, 158.97, 155.74, 135.05, 133.55, 131.25, 130.43, 129.33, 125.27, 119.82, 80.10, 38.55, 31.79, 28.37(3C). HRMS (ESI) exact mass calcd. for $C_{10}H_8NO_2$ $[M+H]^+$ 334.1113, found $[M+H]^+$ 334.1102.

Compound **5** (480 mg, 88% yield) was prepared starting from **1** (500 mg, 3.16 mmol) according to the literature procedure, which was isolated as red solid.[1] ^1H NMR (DMSO) δ 8.4-8.25 (bs, 1H), 8.22-8.09 (s, 1H), 8.04 (d, *J* = 7.7 Hz, 1H), 7.97 (dd, *J* = 7.6, 1.2 Hz, 1H), 7.81 (td, *J* = 7.7, 1.4 Hz, 1H), 7.69 (td, *J* = 7.5, 0.7 Hz, 1H), 5.73 (s, 1H); ^{13}C NMR (DMSO) δ 182.16, 174.62, 157.96, 134.21, 131.61, 131.59, 130.45, 127.73, 123.94, 100.99. HRMS (ESI) exact mass calcd. for $C_{10}H_8NO_2$ $[M+H]^+$ 174.0555, found $[M+H]^+$ 174.0505.

General procedure for the synthesis of 4-methoxy 1,2 naphthoquinone derivatives[2]

1,2 naphthoquinone **1** (200 mg, 1.26mmol) was dissolved in 5 ml of MeOH, in which to it was added $NaIO_3$ (250 mg, 1.26mmol) and $CeCl_3.7H_2O$ (470mg, 1.26mmol) in one portion and stirred vigorously at room temperature. After 20-30 min, the reaction solvent was evaporated under reduced pressure and water and EtOAc were added. The aqueous layer was extracted twice with EtOAc and the combined layers were washed with saturated ammonium chloride. The crude material was purified using column chromatography with Hexanes-EtOAc as eluents to obtain the

product **7** as a yellow solid (160mg, 67% yield). ^{1}H NMR ($CDCl_3$) δ 8.10 (dd, *J* = 7.6, 1.0 Hz, 1H), 7.85 (dd, *J* = 7.8, 0.7 Hz, 1H), 7.68 (td, *J* = 7.7, 1.4 Hz, 1H), 7.57 (td, *J* = 7.6, 1.2 Hz, 1H), 5.97 (s, 1H), 4.01 (s, 3H). ^{13}C NMR ($CDCl_3$) δ 179.65, 179.55, 168.83, 135.11, 132.12, 131.67, 130.51, 129.20, 124.89, 103.21, 56.96. HRMS (ESI) exact mass calcd. for $C_{11}H_9O_3$ $[M+H]^+$ 189.0552, found $[M+H]^+$ 189.0550.

General procedure for the synthesis of 1,2 naphthoquinone derivatives[3]

5-methoxy tetralone (100mg, 0.567 mmol) was dissolved in DMSO (10 mL) and IBX (635 mg, 2.27mmol) was added and heated at 80 ^{o}C for 10-12 hr until the TLC showed the complete disappearance of the starting material. The reaction mixture was then quenched with water and extracted with EtOAc and the combined organic layers were washed with saturated sodium carbonate solution and purified by column using Hexanes-EtOAc as eluents to obtain product **8** as a red solid (69 mg, 65% yield). ^{1}H NMR ($CDCl_3$) δ 7.96 (d, *J* = 10.4 Hz, 1H), 7.69 (d, *J* = 7.6 Hz, 1H), 7.45 (t, *J* = 8.0 Hz, 1H), 7.17 (d, *J* = 8.3 Hz, 1H), 6.34 (d, *J* = 10.4 Hz, 1H), 3.94 (s, 3H). ^{13}C NMR ($CDCl_3$) δ 181.14, 179.55, 156.82, 139.44, 132.97, 132.34, 126.17, 123.20, 122.43, 118.24, 56.34. HRMS (ESI) exact mass calcd. for $C_{11}H_9O_3$ $[M+H]^+$ 189.0552, found $[M+H]^+$ 189.0550.

Compound **9** (80 mg, 75% yield) was prepared starting from 6-methoxy tetralone (100 mg, 0.567 mmol) according to the procedure described above to give the desired product as a red solid. ^{1}H NMR ($CDCl_3$) δ 8.09 (d, *J* = 8.6 Hz, 1H), 7.35 (d, *J* = 10.1 Hz, 1H), 6.93 (dd, *J* = 8.6, 2.4 Hz, 1H), 6.82 (d, *J* = 2.3 Hz, 1H), 6.41 (d, *J* = 10.1 Hz, 1H), 3.92 (s, 3H). ^{13}C NMR ($CDCl_3$) δ 181.67, 177.43, 165.84, 144.93, 137.07, 133.37, 128.76, 125.15, 116.09, 114.90, 56.07. HRMS (ESI) exact mass calcd. for $C_{11}H_9O_3$ $[M+H]^+$ 189.0552, found $[M+H]^+$ 189.0578.

Compound **10** (73mg, 68% yield) was prepared starting from 7-methoxy tetralone (100 mg, 0.567 mmol) according to the procedure described above give the desired product as a red solid. ^{1}H NMR ($CDCl_3$) δ 7.58 (d, *J* = 2.7 Hz, 1H), 7.36 (d, *J* = 10.1 Hz, 1H), 7.24 (d, *J* = 1.1 Hz, 1H), 7.10 (dd, *J* = 8.4, 2.7 Hz, 1H), 6.26 (d, *J* = 10.1 Hz, 1H), 3.88 (s, 3H). ^{13}C NMR ($CDCl_3$) δ 181.09, 179.12, 162.00, 145.70, 133.29, 131.70, 128.05, 125.28, 121.81, 114.87, 56.03. HRMS (ESI) exact mass calcd. for $C_{11}H_9O_3$ $[M+H]^+$ 189.0552, found $[M+H]^+$ 189.0550.

General procedure for the synthesis of di-substituted 1,2 naphthoquinones

6-methoxy tetralone (100mg, 0.567 mmol) was dissolved in DMSO (10 mL) and IBX (635 mg, 2.27mmol) was added and heated at 80 ^{o}C for 10-12h until the TLC showed the complete

disappearance of starting material. The reaction mixture was then quenched with water and extracted with EtOAc and the combined organic layers were washed with saturated sodium carbonate solution. The crude material was then dissolved in 5 ml of MeOH and $NaIO_3$ (112 mg, 0.567 mmol) and $CeCl_3.7H_2O$ (470mg, 0.567 mmol) were added in one portion and stirred vigorously at room temperature. After 20-30 minutes, solvent was evaporated under reduced pressure followed by the addition of water and EtOAc. The crude material was purified using $CHCl_3$/EtOAc as eluents to give as **12** as a yellow solid (43mg, 35% yield over two steps). ^1H NMR ($CDCl_3$) δ 8.07 (d, J = 8.6 Hz, 1H), 7.30 (d, J = 2.5 Hz, 1H), 7.00 (dd, J = 8.6, 2.5 Hz, 1H), 5.93 (s, 1H), 3.99 (s, 3H), 3.93 (s, 3H). ^{13}C NMR ($CDCl_3$) δ 180.24, 178.12, 168.05, 165.25, 134.40, 132.06, 123.92, 116.24, 110.64, 103.55, 56.87, 56.05. HRMS (ESI) exact mass calcd. for $C_{12}H_{11}O_4$ $[M+H]^+$ 219.0657, found $[M+H]^+$ 219.0629.

Compound **13** (38 mg, 31% yield over two steps) was prepared starting from 7-methoxy tetralone (100 mg, 0.567 mmol) according to the procedure described above which was obtained as a red solid. ^1H NMR ($CDCl_3$) δ 7.76 (d, J = 8.7 Hz, 1H), 7.60 (d, J = 2.7 Hz, 1H), 7.16 (dd, J = 8.7, 2.8 Hz, 1H), 5.87 (s, 1H), 4.00 (s, 3H), 3.91 (s, 3H). ^{13}C NMR ($CDCl_3$) δ 179.80, 179.76, 169.60, 162.47, 132.20, 126.74, 124.88, 121.34, 113.22, 101.29, 56.86, 56.02. HRMS (ESI) exact mass calcd. for $C_{12}H_{11}O_4$ $[M+H]^+$ 219.0657, found $[M+H]^+$ 219.0617.

Compound **14** (43 mg, 38% yield over two steps) was prepared starting from 6-OTosyl tetralone (100 mg, 0.316 mmol) according to the procedure described above as yellow solid. ^1H NMR ($CDCl_3$) δ 8.02 (d, J = 8.4 Hz, 1H), 7.75 (d, J = 8.3 Hz, 2H), 7.66 (d, J = 2.3 Hz, 1H), 7.36 (d, J = 8.0 Hz, 2H), 7.04 (dd, J = 8.4, 2.3 Hz, 1H), 5.99 (s, 1H), 4.01 (s, 3H), 2.47 (s, 3H); ^{13}C NMR ($CDCl_3$) δ 179.01, 178.28, 167.22, 154.60, 146.35, 134.35, 132.06, 131.16, 130.24 (2C), 128.83, 128.63 (2C), 124.80, 119.38, 104.05, 57.15, 21.92. HRMS (ESI) exact mass calcd. for $C_{18}H_{15}O_6S$ $[M+H]^+$ 359.0589, found $[M+H]^+$ 359.0549.

Synthesis of 3-hydroxy lapachone (**25**) [4]

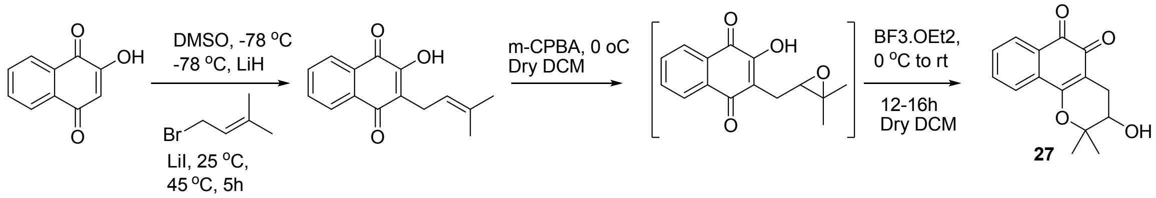

Compound **25** was prepared according to the literature procedure**:** ^{1}H NMR ($CDCl_3$) δ 8.04 (dd, *J* = 7.6, 1.0 Hz, 1H), 7.83 (d, *J* = 7.3 Hz, 1H), 7.65 (td, *J* = 7.7, 1.3 Hz, 1H), 7.51 (td, *J* = 7.6, 1.0 Hz, 1H), 3.93 (t, *J* = 5.1 Hz, 1H), 2.81 (dd, *J* = 17.7, 4.9 Hz, 1H), 2.62 (dd, *J* = 17.7, 5.3 Hz, 1H), 2.15 (d, *J* = 9.5 Hz, 1H), 1.51 (s, 3H), 1.45 (s, 3H). ^{13}C NMR ($CDCl_3$) δ 179.66, 178.88, 161.67, 135.02, 132.22, 131.07, 130.20, 128.88, 124.51, 110.56, 81.62, 68.42, 25.52, 25.23, 22.23. HRMS (ESI) exact mass calcd. for $C_{15}H_{15}O_4$ $[M+H]^+$ 259.0970, found $[M+H]^+$ 259.0954.

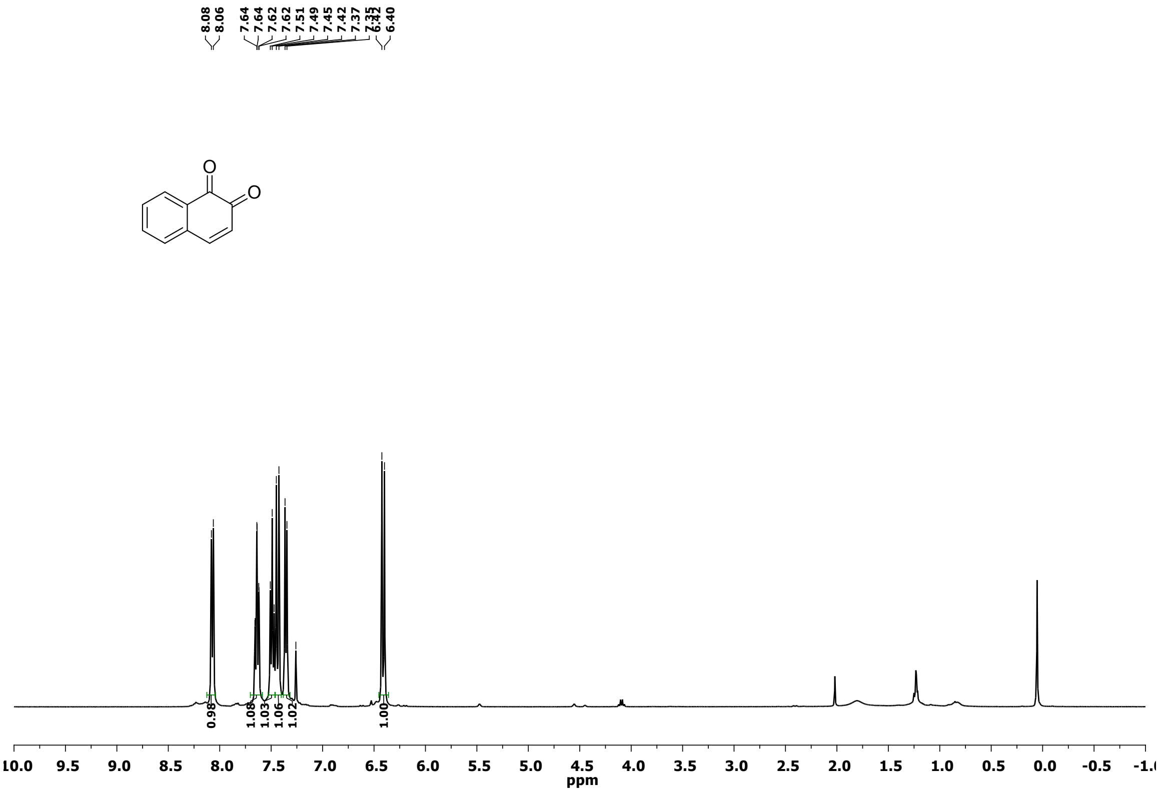
8.08
8.06
7.64
7.64
7.62
7.62
7.51
7.49
7.45
7.42
7.37
7.35
6.42
6.40
0.98
1.08
1.03
1.06
1.02
1.00
ppm

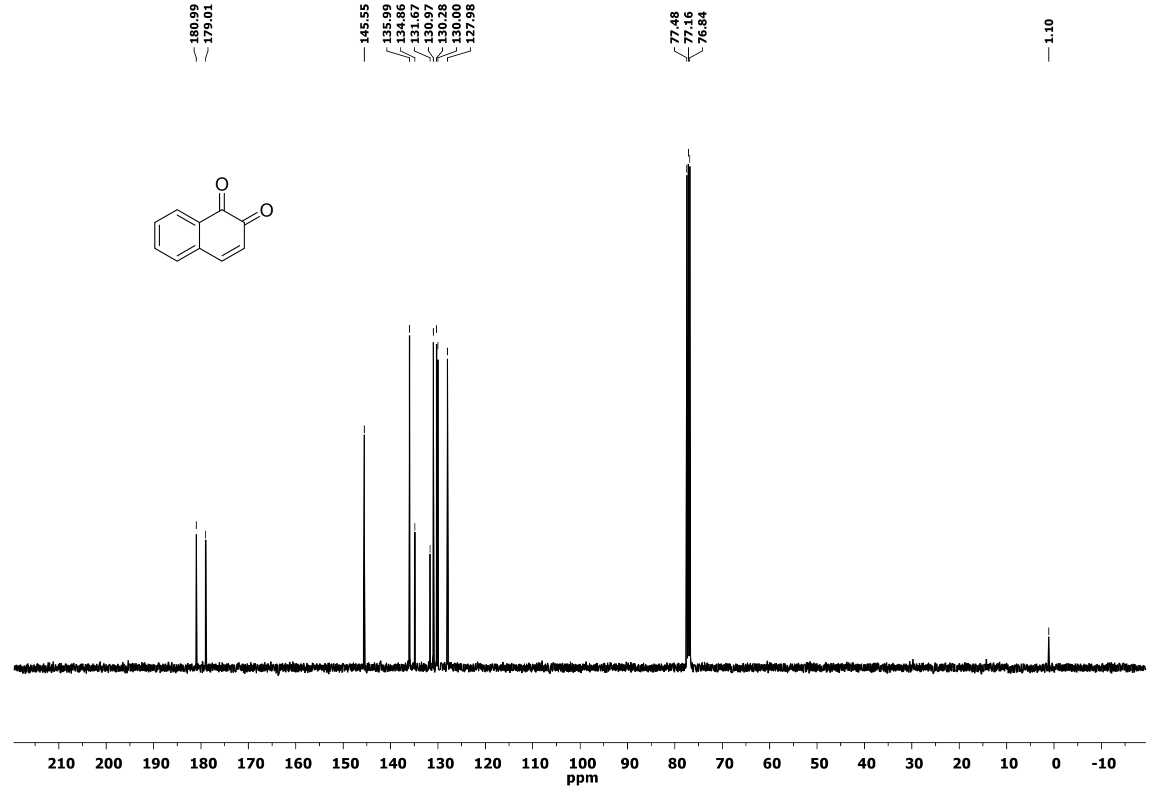
180.99
179.01
145.55
135.99
134.86
131.67
130.97
130.28
130.00
127.98
77.48
77.16
76.84
1.10
ppm

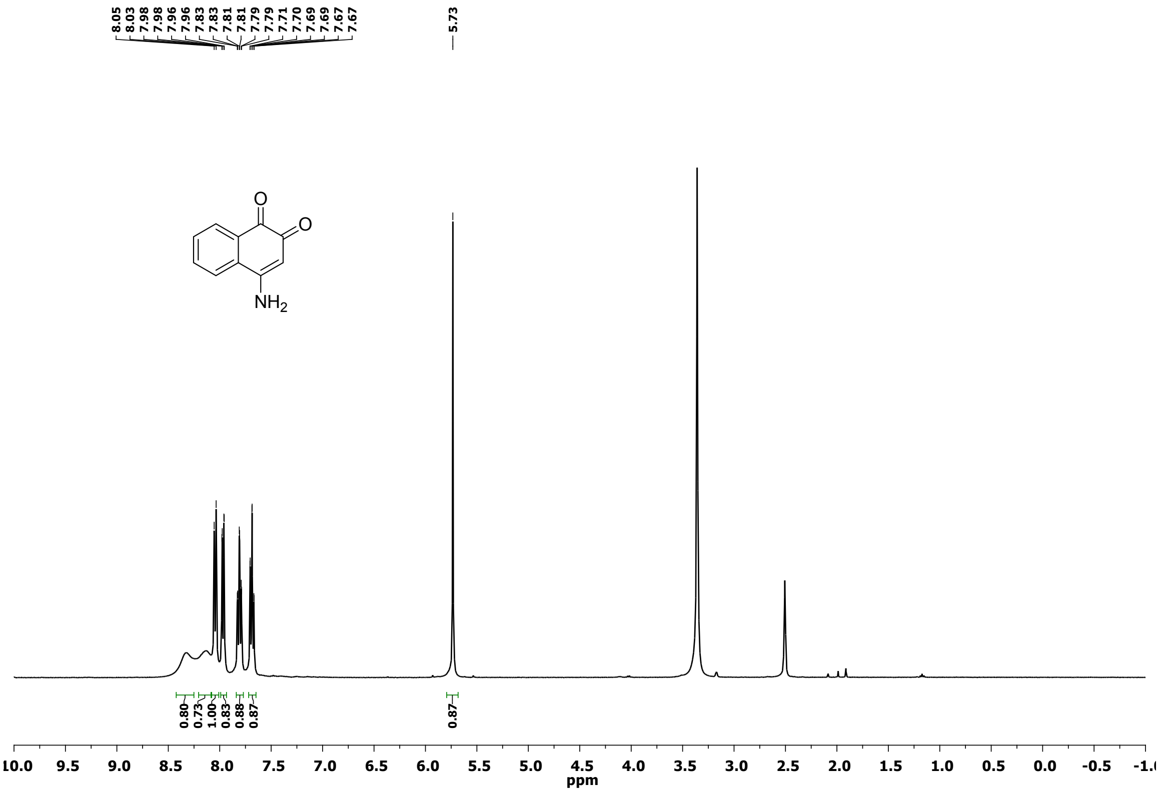

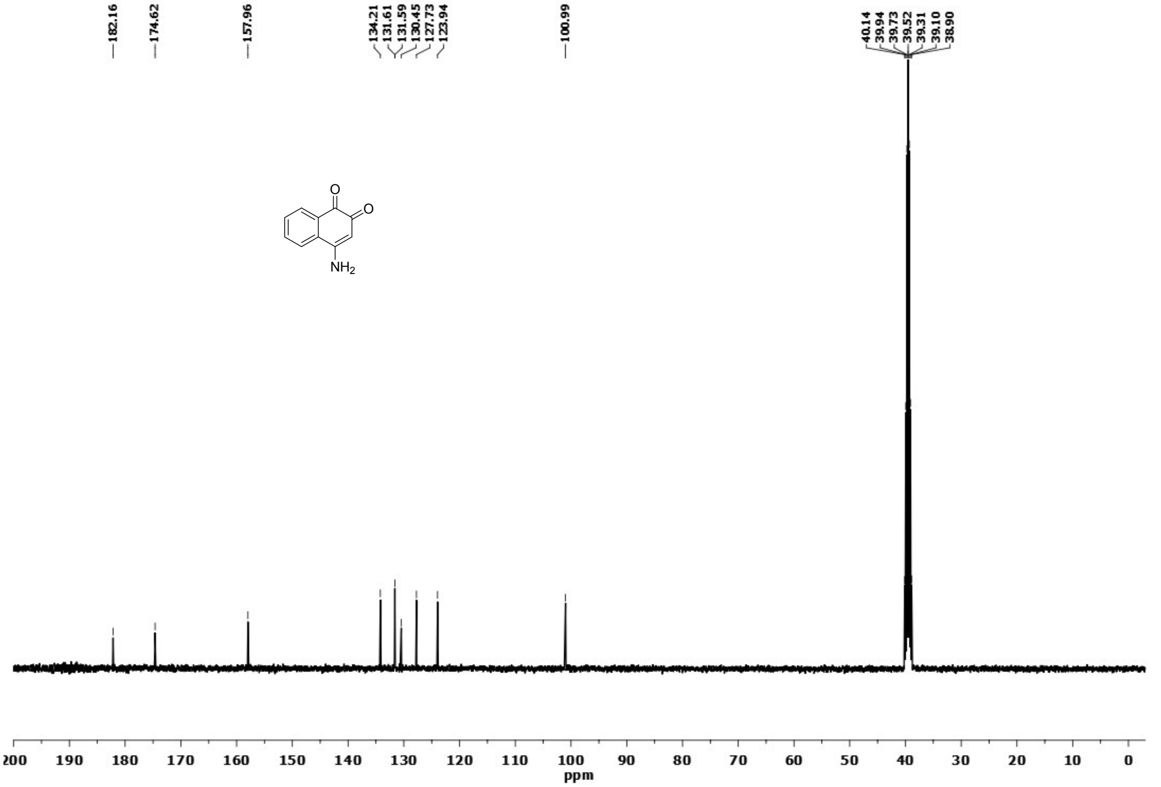

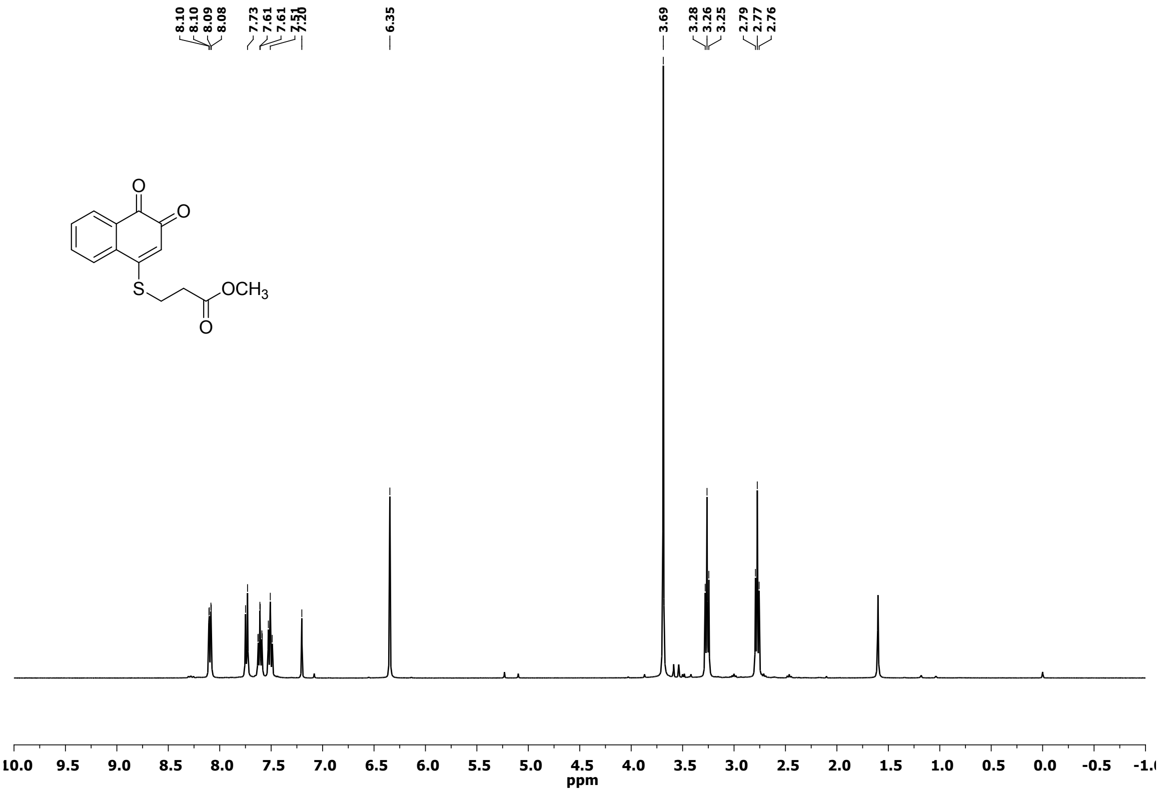
8.10
8.10
8.09
8.08
7.73
7.61
7.61
7.51
7.20
6.35
3.69
3.28
3.26
3.25
2.79
2.77
2.76
O
O
S
OCH3
O
10.0 9.5 9.0 8.5 8.0 7.5 7.0 6.5 6.0 5.5 5.0 4.5 4.0 3.5 3.0 2.5 2.0 1.5 1.0 0.5 0.0 -0.5
ppm

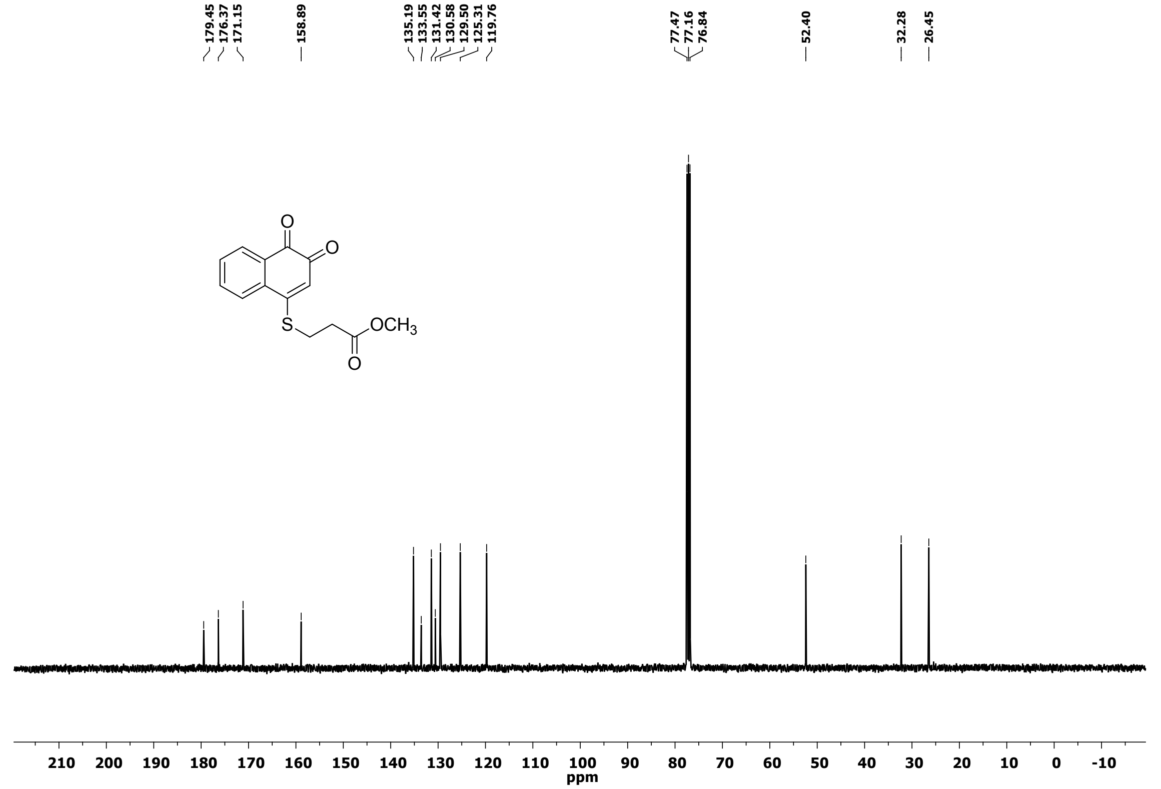
179.45
176.37
171.15
158.89
135.19
133.55
131.42
130.58
129.50
125.31
119.76
77.47
77.16
76.84
52.40
32.28
26.45
O
O
S
OCH3
O
210 200 190 180 170 160 150 140 130 120 110 100 90 80 70 60 50 40 30 20 10 0 -10
ppm

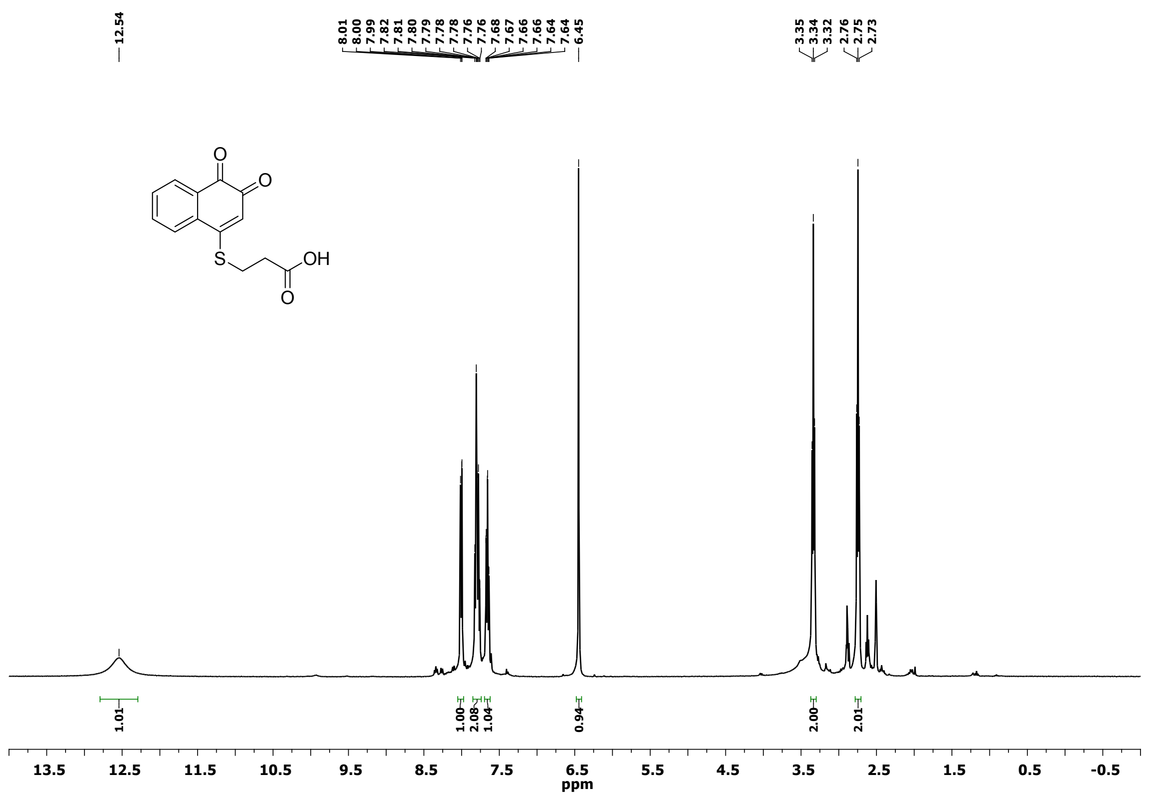

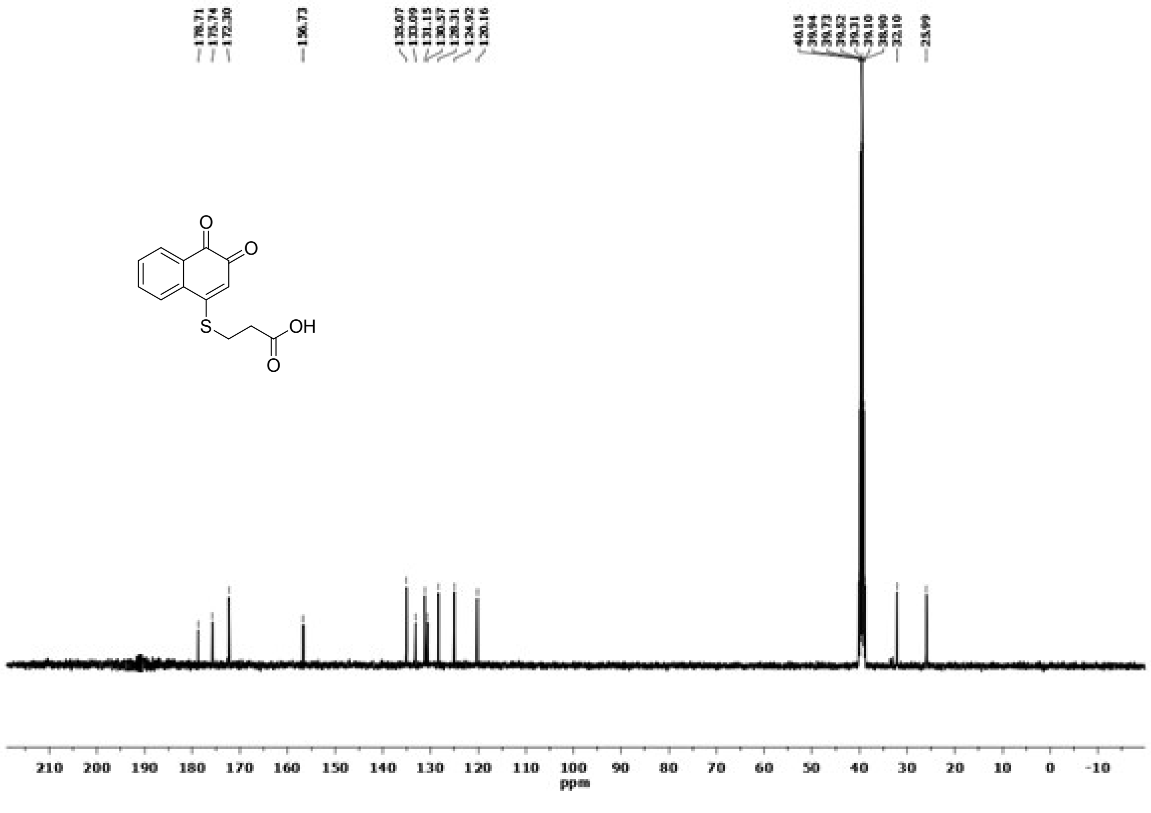

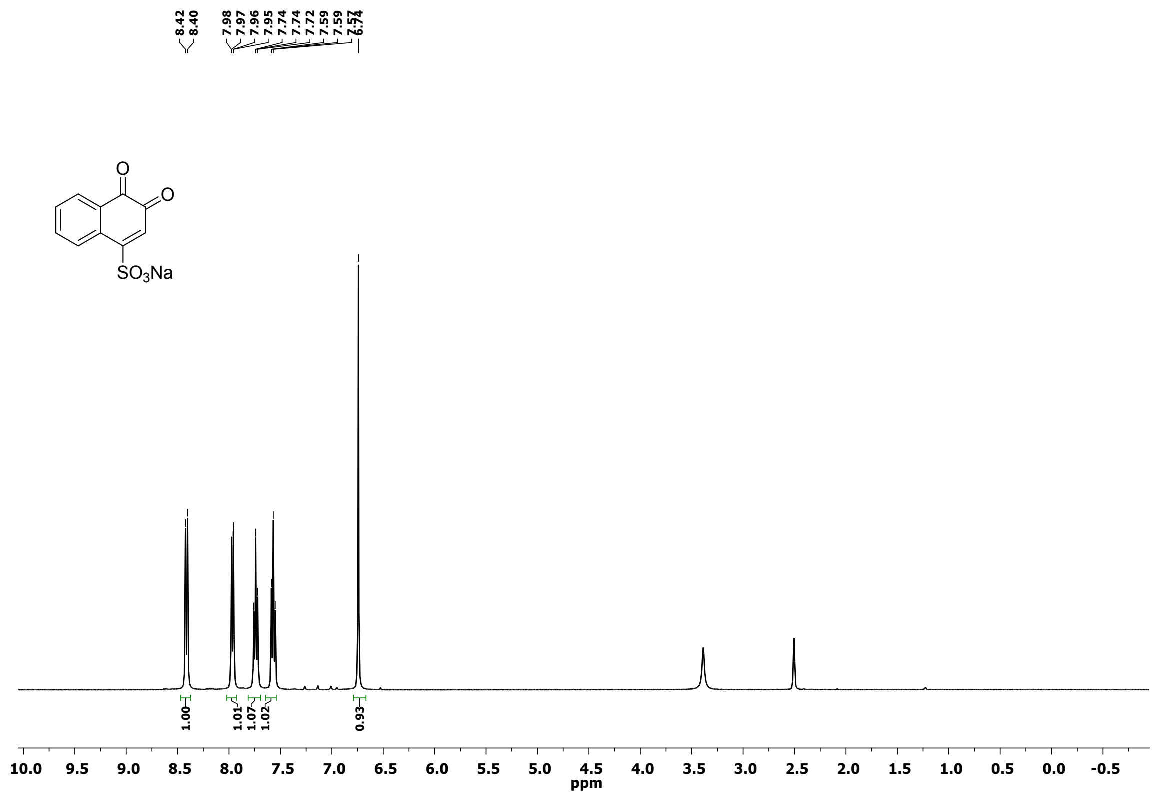
8.42
8.40
7.98
7.97
7.96
7.95
7.74
7.74
7.72
7.59
7.59
7.57
6.74
SO3Na
1.00
1.01
1.07
1.02
0.93
10.0 9.5 9.0 8.5 8.0 7.5 7.0 6.5 6.0 5.5 5.0 4.5 4.0 3.5 3.0 2.5 2.0 1.5 1.0 0.5 0.0 -0.5
ppm

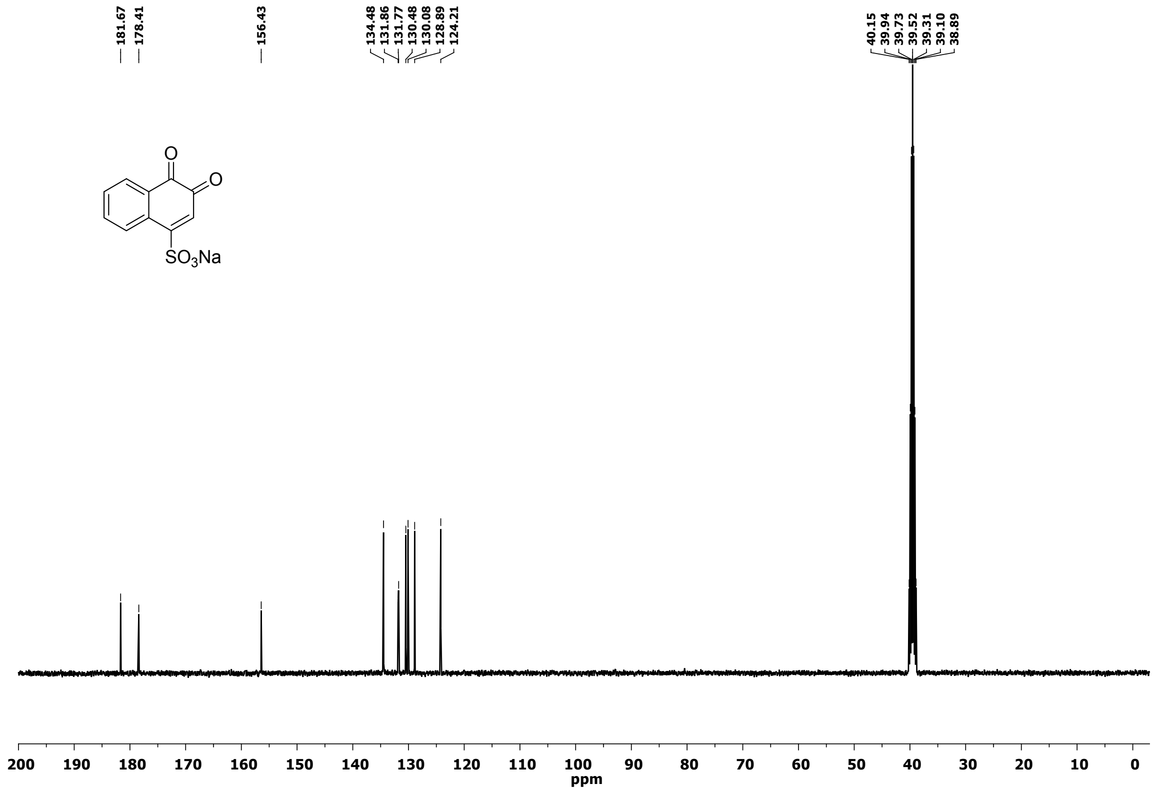
181.67
178.41
156.43
134.48
131.86
131.77
130.48
130.08
128.89
124.21
40.15
39.94
39.73
39.52
39.31
39.10
38.89
SO3Na
200 190 180 170 160 150 140 130 120 110 100 90 80 70 60 50 40 30 20 10 0
ppm

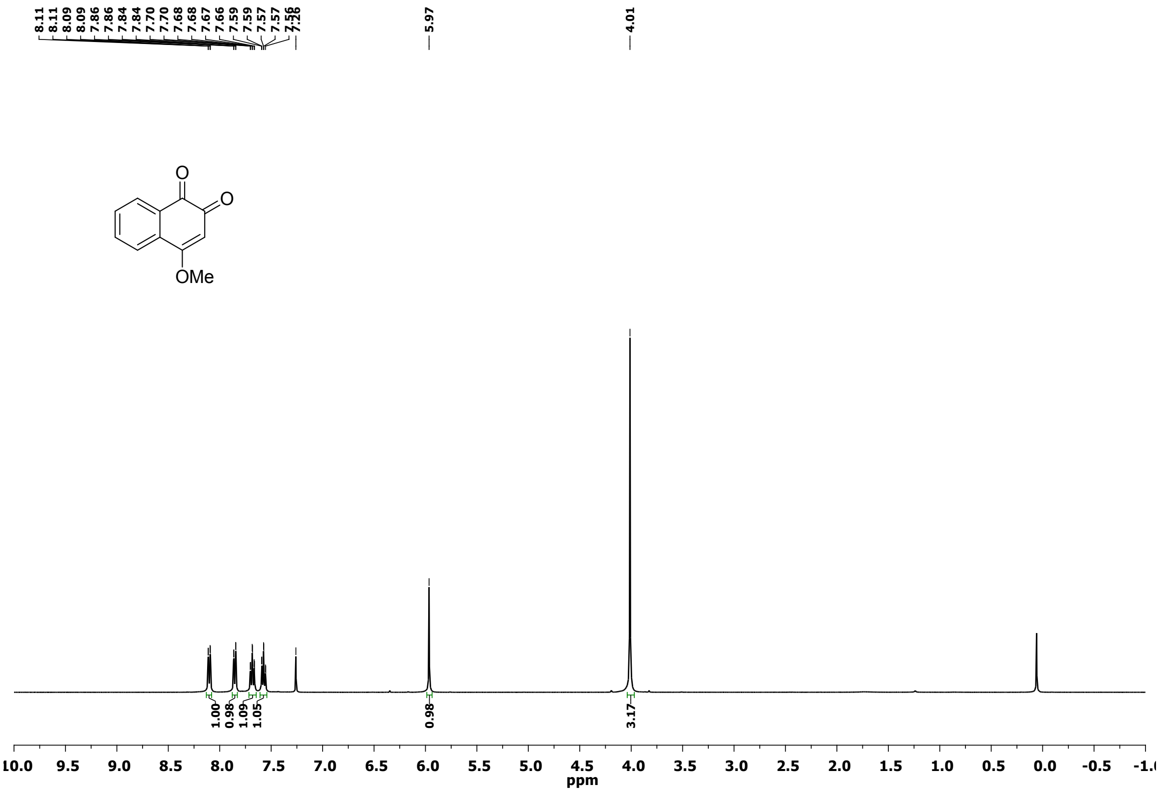
8.11
8.11
8.09
8.09
7.86
7.86
7.84
7.84
7.70
7.70
7.68
7.68
7.67
7.66
7.59
7.59
7.57
7.57
5.97
4.01
O
O
OMe
1.00
0.98
1.09
1.05
0.98
3.17
10.0 9.5 9.0 8.5 8.0 7.5 7.0 6.5 6.0 5.5 5.0 4.5 4.0 3.5 3.0 2.5 2.0 1.5 1.0 0.5 0.0 -0.5
ppm

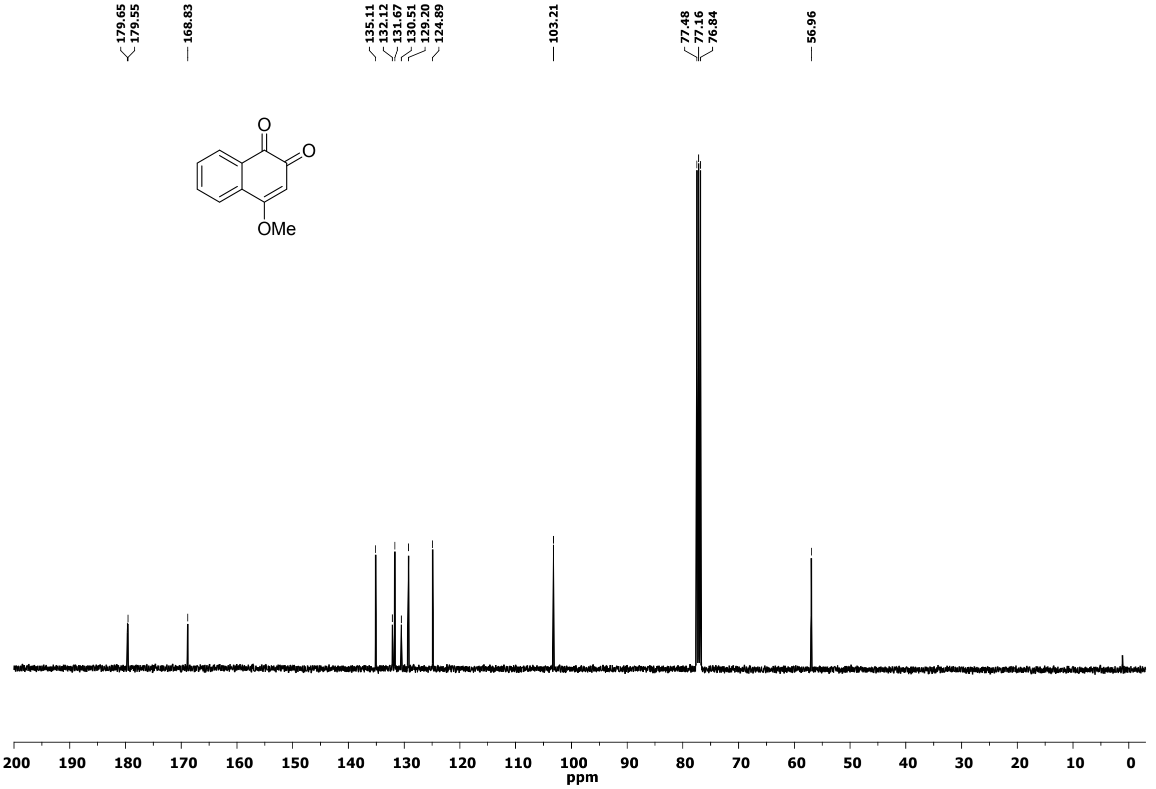
179.65
179.55
168.83
135.11
132.12
131.67
130.51
129.20
124.89
103.21
77.48
77.16
76.84
56.96
O
O
OMe
200 190 180 170 160 150 140 130 120 110 100 90 80 70 60 50 40 30 20 10 0
ppm

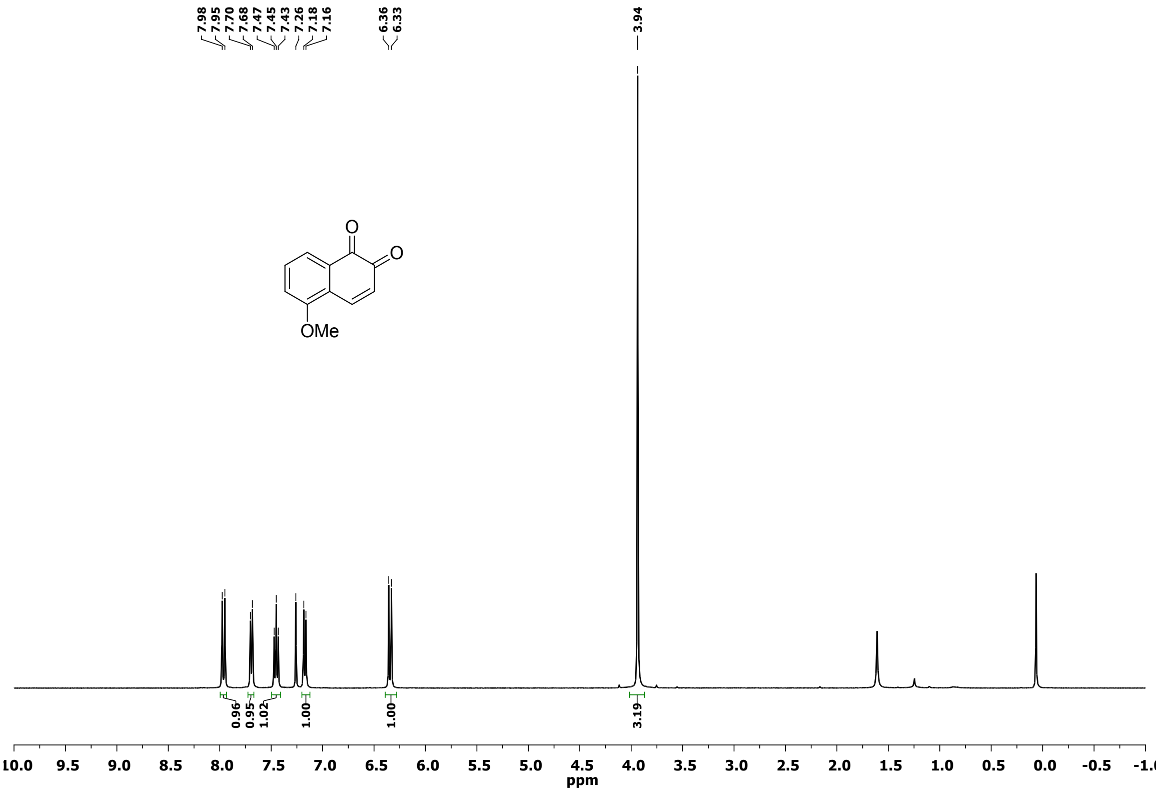
7.98
7.95
7.70
7.68
7.47
7.45
7.43
7.26
7.18
7.16
6.36
6.33
3.94
OMe
0.96
0.95
1.02
1.00
1.00
3.19
ppm

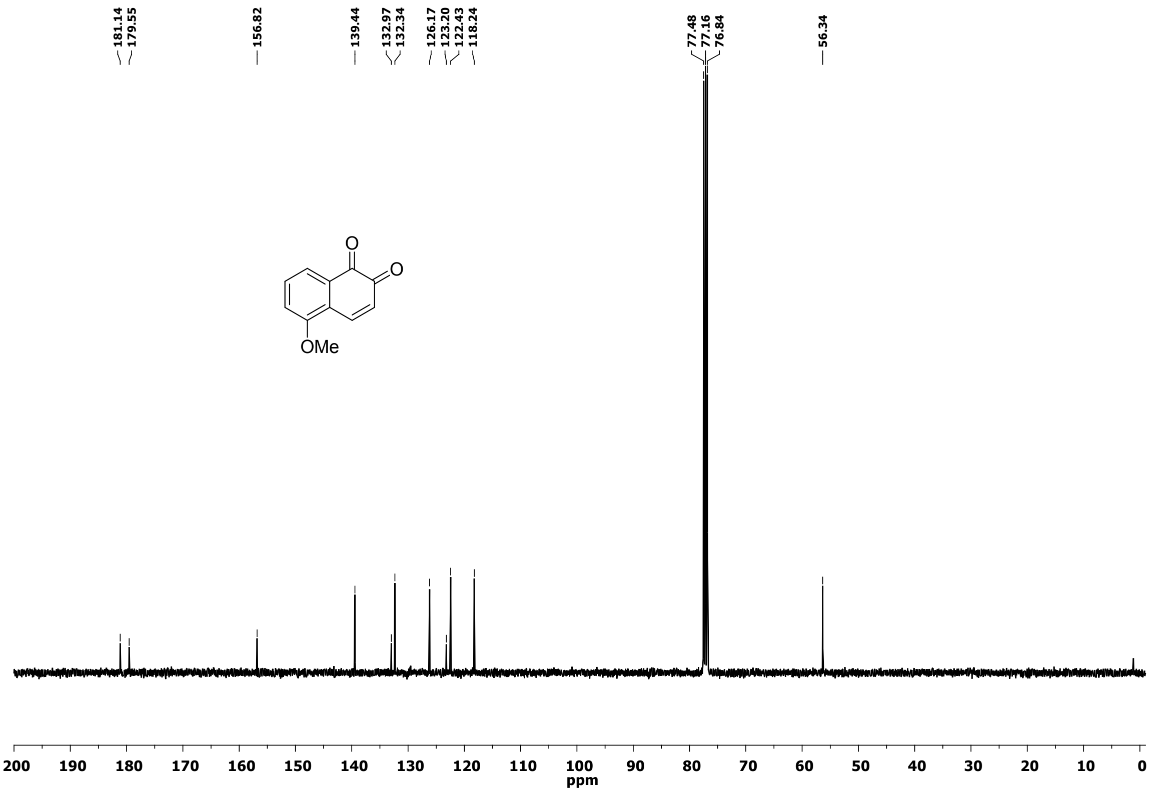
181.14
179.55
156.82
139.44
132.97
132.34
126.17
123.20
122.43
118.24
77.48
77.16
76.84
56.34
OMe
ppm

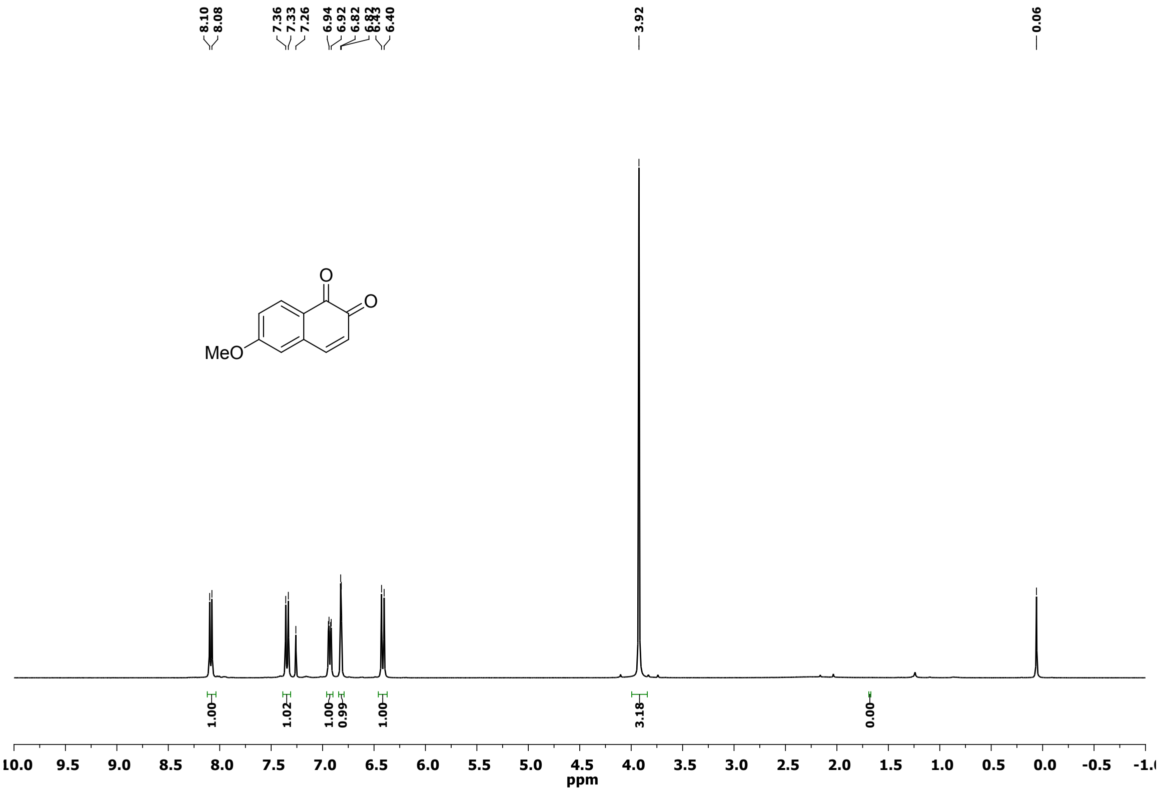
8.10
8.08
7.36
7.33
7.26
6.94
6.92
6.82
6.43
6.40
3.92
0.06
MeO
O
O
1.00
1.02
1.00
0.99
1.00
3.18
0.00
10.0 9.5 9.0 8.5 8.0 7.5 7.0 6.5 6.0 5.5 5.0 4.5 4.0 3.5 3.0 2.5 2.0 1.5 1.0 0.5 0.0 -0.5
ppm

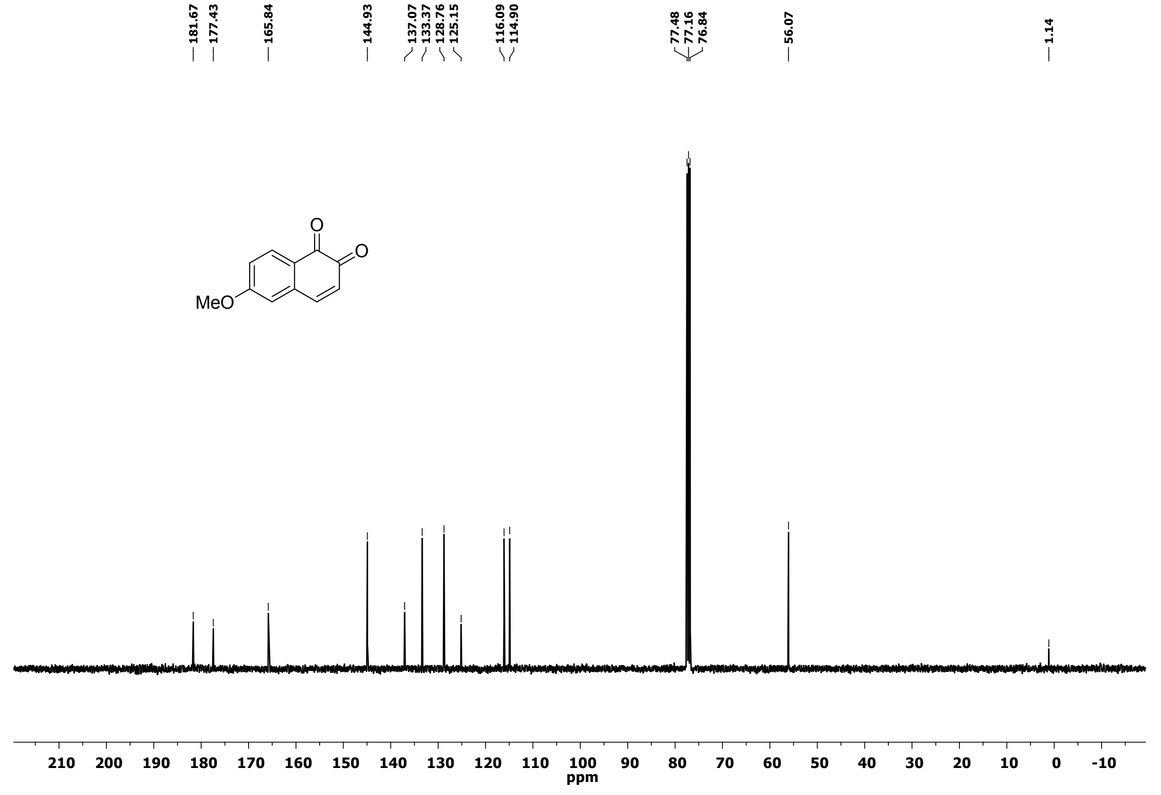
181.67
177.43
165.84
144.93
137.07
133.37
128.76
125.15
116.09
114.90
77.48
77.16
76.84
56.07
1.14
MeO
O
O
210 200 190 180 170 160 150 140 130 120 110 100 90 80 70 60 50 40 30 20 10 0 -10
ppm

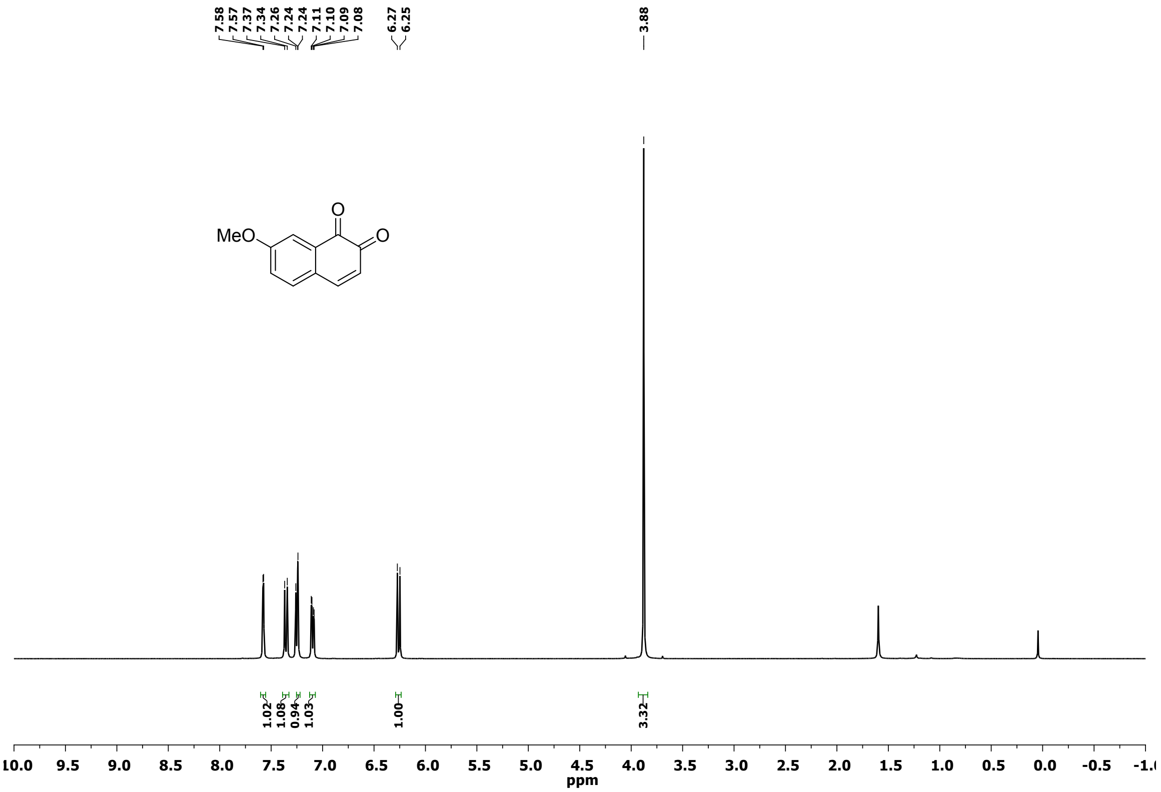
7.58
7.57
7.37
7.34
7.26
7.24
7.24
7.11
7.10
7.09
7.08
6.27
6.25
3.88
MeO
O
O
1.02
1.08
0.94
1.03
1.00
3.32
10.0 9.5 9.0 8.5 8.0 7.5 7.0 6.5 6.0 5.5 5.0 4.5 4.0 3.5 3.0 2.5 2.0 1.5 1.0 0.5 0.0 -0.5
ppm

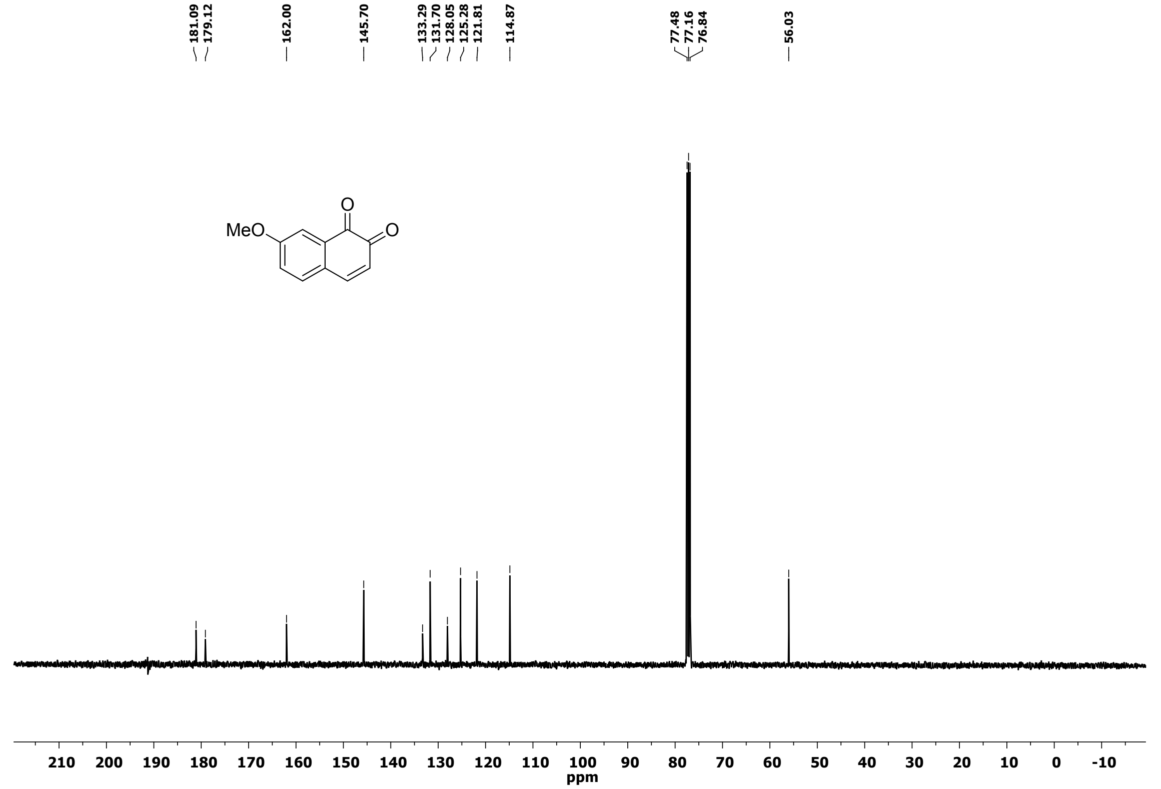
181.09
179.12
162.00
145.70
133.29
131.70
128.05
125.28
121.81
114.87
77.48
77.16
76.84
56.03
MeO
O
O
210 200 190 180 170 160 150 140 130 120 110 100 90 80 70 60 50 40 30 20 10 0 -10
ppm

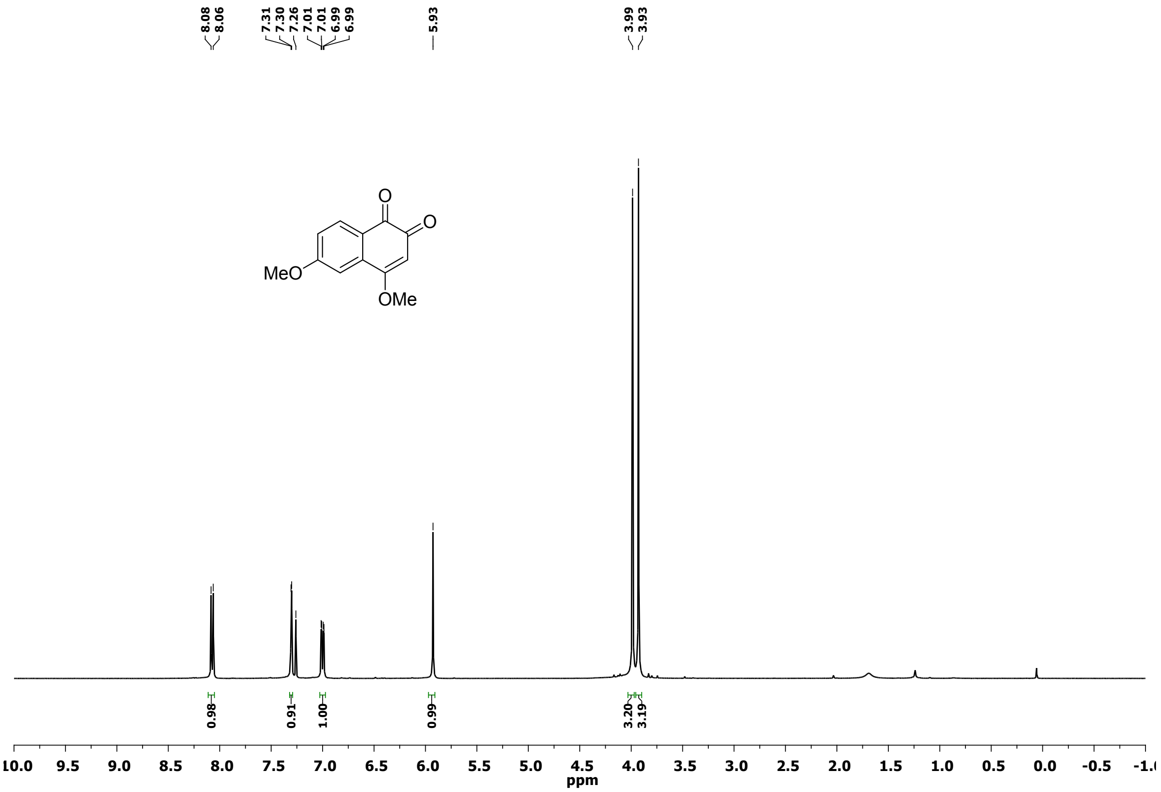

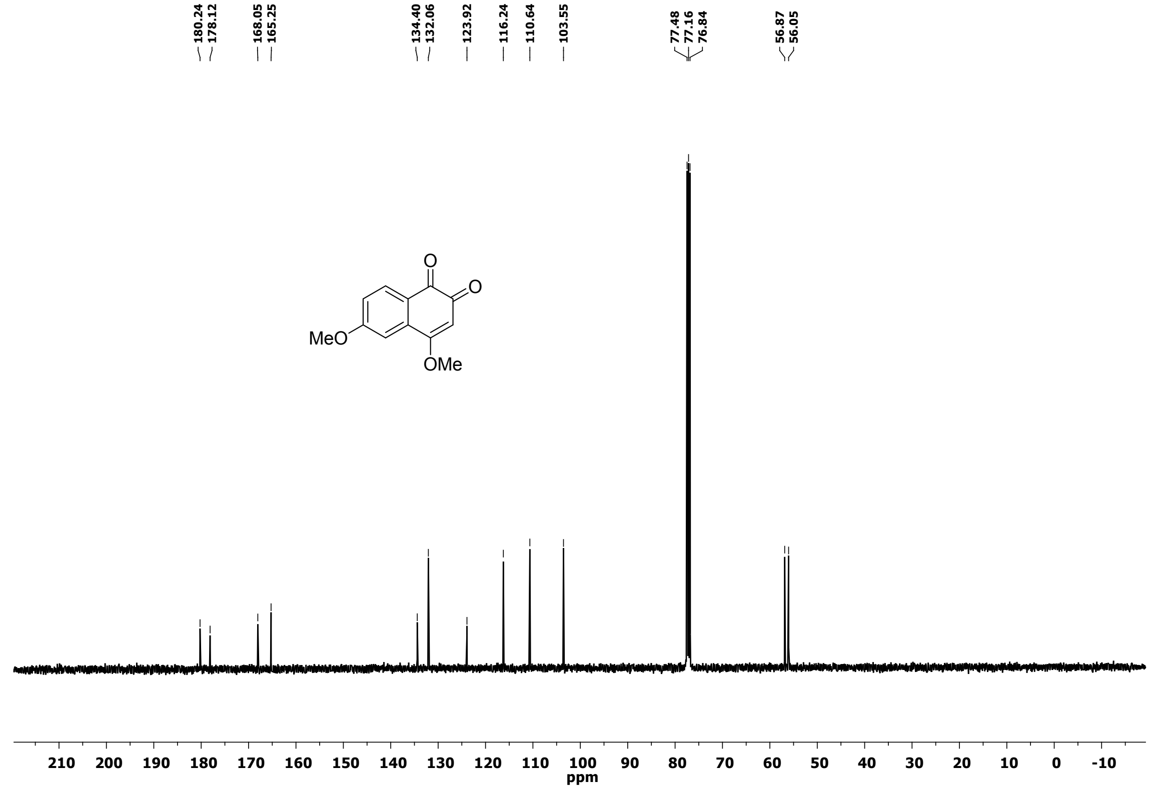

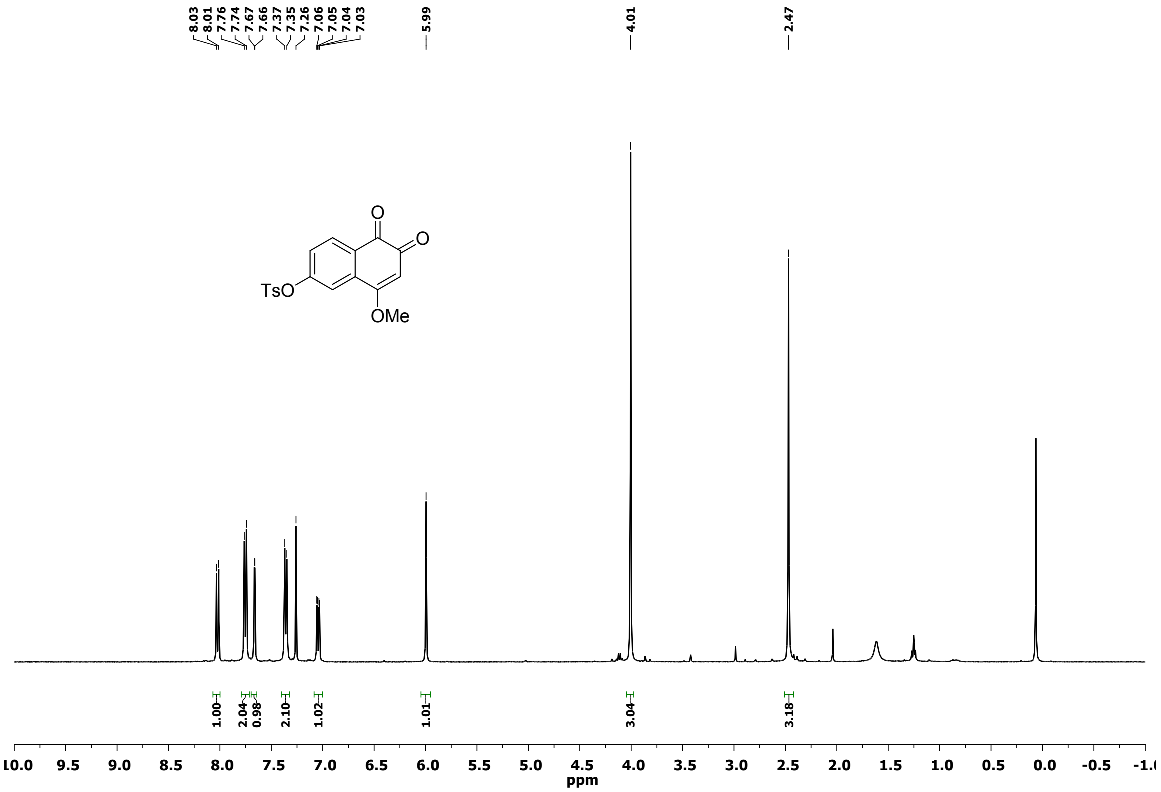

8.03
8.01
7.76
7.74
7.67
7.66
7.37
7.35
7.26
7.06
7.05
7.04
7.03
5.99
4.01
2.47
TsO
O
O
OMe
1.00
2.04
0.98
2.10
1.02
1.01
3.04
3.18
10.0 9.5 9.0 8.5 8.0 7.5 7.0 6.5 6.0 5.5 5.0 4.5 4.0 3.5 3.0 2.5 2.0 1.5 1.0 0.5 0.0 -0.5 -1.0
ppm

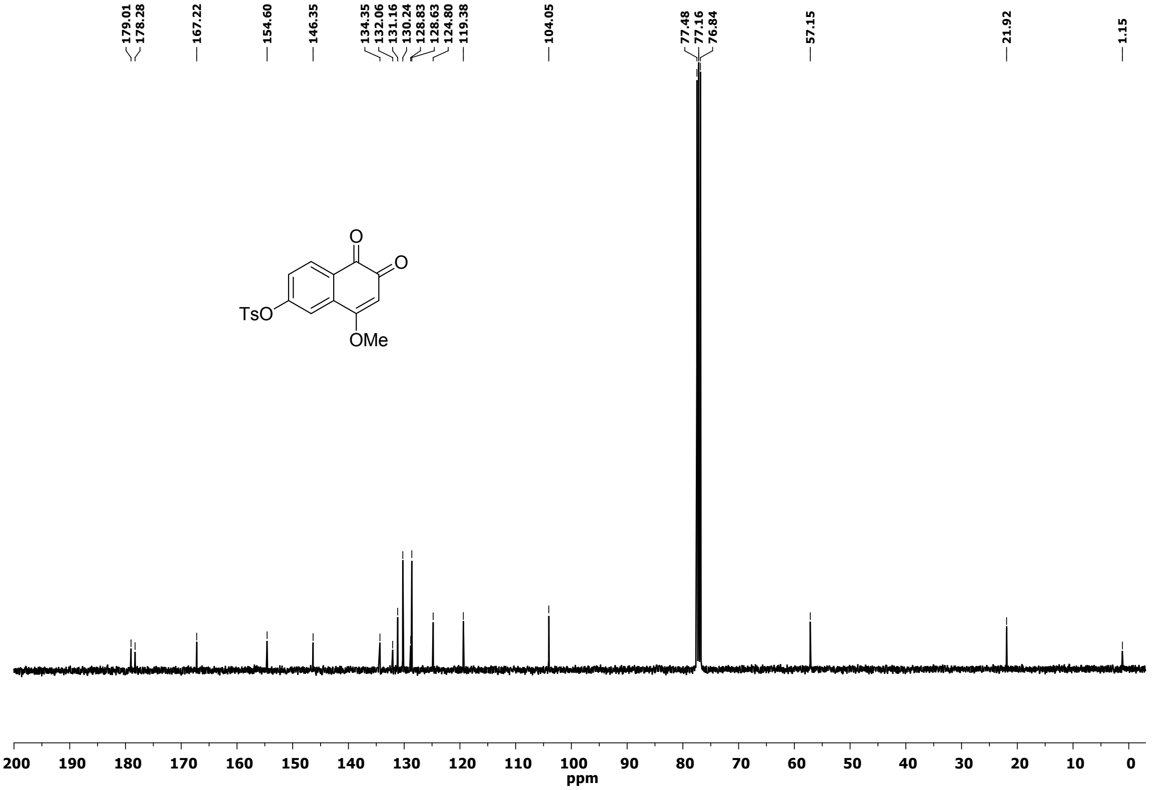

179.01
178.28
167.22
154.60
146.35
134.35
132.06
131.16
130.24
128.83
128.63
124.80
119.38
104.05
77.48
77.16
76.84
57.15
21.92
1.15
TsO
O
O
OMe
200 190 180 170 160 150 140 130 120 110 100 90 80 70 60 50 40 30 20 10 0
ppm

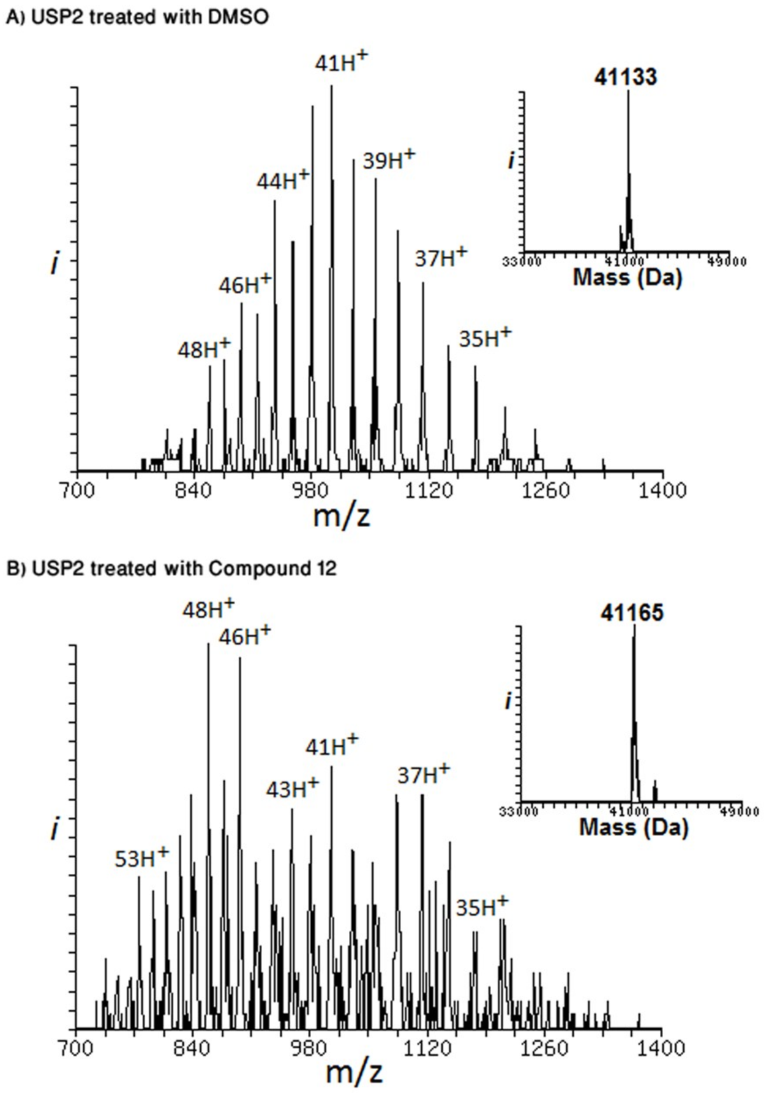

Figure 1: Mass spectrometry (ESI-MS) of USP2 treated with a) DMSO (Observed mass - 41133 Da) and b) Compound **12** for 15 mts (Observed mass - 41165 Da).

Cell culture:

DU-145 cells were grown in EMEM medium supplemented with 10% fetal bovine serum, 100 units/ml penicillin and 100 mg/ml streptomycin in 37 ^{0}C humidified incubator with a 5% CO_2, 95% air atmosphere.

Apoptosis studies:

Induction of apoptosis in DU-145 cell line by treatment with **7**, **9**, **12** and **18** were determined after 2 h incubation in a dose dependent manner, using annexin V-FITC apoptosis detection kit

(BD Biosciences) according to the manufacturer`s protocol and monitored via flow-cytometry (fluorescence-activated cell sorting, FACS). Briefly, 2 x 10^5 cells/well were seeded in 6-well plates and treated with inhibitor for 2 hr in a dose dependent manner. The cells were then harvested and washed with PBS. Next, the cells were re-suspended with 85 μL binding buffer and stained with 10 μL annexin V-FITC reagent and 5 μL propidium iodide (PI) for 15 min in the dark. The increase in fluorescence, which indicates the apoptosis level in the treated cells, were monitored using flow cytometry and compared to untreated cells containing DMSO as a control.

References:

1. L. F. Fieser and J. L. Hartwell, *J. Am. Chem. Soc.*, 1935, **57**, 1482-1484.
2. A. Takuwa, O. Soga, H. Iwamoto and K. Maruyama, *Bull. Chem. Soc. Jpn.*, 1986, **59**, 2959-2961.
3. J. Ren, L. Lu, J. Xu, T. Yu and B.-B. Zeng, *Synthesis*, 2015, **47**, 2270-2280.
4. J. S. Sun, A. H. Geiser and B. Frydman, *Tetrahedron Lett.*, 1998, **39**, 8221-8224.
